# Supplementary material for: A lung tropic AAV vector improves survival in a mouse model of surfactant B deficiency
Source: Nat Commun. 2020 Aug 6;11:3929. doi: 10.1038/s41467-020-17577-8 (PMC7414154; doi:10.1038/s41467-020-17577-8)
Supplement: Supplementary file 13 — Reporting Summary [file 41467_2020_17577_MOESM13_ESM.pdf]

nature research

Corresponding author(s): Drs. Bernard Thebaud and Sarah K Wootton

Last updated by author(s): May 25, 2020

Reporting Summary

Nature Research wishes to improve the reproducibility of the work that we publish. This form provides structure for consistency and transparency in reporting. For further information on Nature Research policies, see our Editorial Policies and the Editorial Policy Checklist.

Statistics

For all statistical analyses, confirm that the following items are present in the figure legend, table legend, main text, or Methods section.

n/a	Confirmed	
☐	☒	The exact sample size (*n*) for each experimental group/condition, given as a discrete number and unit of measurement
☐	☒	A statement on whether measurements were taken from distinct samples or whether the same sample was measured repeatedly
☐	☒	The statistical test(s) used AND whether they are one- or two-sided<br>*Only common tests should be described solely by name; describe more complex techniques in the Methods section.*
☒	☐	A description of all covariates tested
☐	☒	A description of any assumptions or corrections, such as tests of normality and adjustment for multiple comparisons
☐	☒	A full description of the statistical parameters including central tendency (e.g. means) or other basic estimates (e.g. regression coefficient) AND variation (e.g. standard deviation) or associated estimates of uncertainty (e.g. confidence intervals)
☐	☒	For null hypothesis testing, the test statistic (e.g. *F*, *t*, *r*) with confidence intervals, effect sizes, degrees of freedom and *P* value noted<br>*Give P values as exact values whenever suitable.*
☒	☐	For Bayesian analysis, information on the choice of priors and Markov chain Monte Carlo settings
☒	☐	For hierarchical and complex designs, identification of the appropriate level for tests and full reporting of outcomes
☒	☐	Estimates of effect sizes (e.g. Cohen's *d*, Pearson's *r*), indicating how they were calculated

Our web collection on statistics for biologists contains articles on many of the points above.

Software and code

Policy information about availability of computer code

Data collection	IVIS images were quantified using the Living Image 3.2 software. Sample compensation in the flow cytometry study was performed with the BD FACSDiva version 8.0 software. All lung function data from the flexiVent was obtained using flexiWare version 7.0. RT-qPCR Ct data from lung tissue was obtained using the StepOnePlus software 2.3. Scanned brightfield images using Aperio's CS2 digital brightfield scanner was processed using Aperio's ImageScope version 12.3 software. Fluorescence microscopy images were processed using the AxioVision 40x64 version 4.9.1.0 application. Confocal microscope images were processed using the Zen 2 (Blue edition) software. Data from mouse inflammatory cytokine measurements using flow cytometry was collected using BD FACSDiva software version 8.0 (firmware 1.49).
Data analysis	All statistical analyses was carried out using Prism8 for macOS (Version 8.3.0).

For manuscripts utilizing custom algorithms or software that are central to the research but not yet described in published literature, software must be made available to editors and reviewers. We strongly encourage code deposition in a community repository (e.g. GitHub). See the Nature Research guidelines for submitting code & software for further information.

Data

Policy information about availability of data

All manuscripts must include a data availability statement. This statement should provide the following information, where applicable:

- Accession codes, unique identifiers, or web links for publicly available datasets
- A list of figures that have associated raw data
- A description of any restrictions on data availability

The authors declare that the data supporting the findings of this study are available within the paper, the associated Supplementary Figures and Tables, and in the Source Data files. The source data underlying Figs. 2c-d, Fig. 3b, Fig. 4g, Fig. 6h, Fig. 7h, and Fig. 10f are provided as Source Data files. All other information is freely available from the corresponding authors upon request.

Field-specific reporting

Please select the one below that is the best fit for your research. If you are not sure, read the appropriate sections before making your selection.

☒ Life sciences ☐ Behavioural & social sciences ☐ Ecological, evolutionary & environmental sciences

For a reference copy of the document with all sections, see nature.com/documents/nr-reporting-summary-flat.pdf

Life sciences study design

All studies must disclose on these points even when the disclosure is negative.

Sample size	In the majority of experiments we chose 10 animals per treatment group as the sample size. A sample size of 10 was arbitrarily chosen for our first survival study using the low dose of AAV-mSPB (10^10vg/mouse). Because there was a statistically significant improvement in median survival using n=10 with this low dose of AAV-mSPB, we chose to continue using this sample size for all our structure/function as well as survival studies in which a higher dose of AAV-SPB was used in all cases. However, in some instances the sample size per group was larger (n=11) or smaller (n=5-9) based on animal availability.
Data exclusions	No data was excluded from this study. All experiments related to this study are included in the manuscript or in the Supplementary Figures.
Replication	In all survival as well as structure and function experiments, we used an inducible compound transgenic SP-B deficient mouse model. We performed a total of 9 survival studies with AAV-murine SPB under various conditions including using different AAV dosages, different administration methods, with or without exogenous pulmonary surfactant as a vehicle (BLES), and different times of dox removal. We independently replicated the survival study in which an intermediate dose of AAV-mSPB (10^11vg/mouse) was administered with dox removal 29 days later (Supplementary Fig 6c and Fig 6b). In the initial study (Supplementary Fig 6c) median survival was 9 days and in the replication study (Fig 6b) median survival was 20 days demonstrating an improvement in median survival. We also independently replicated the survival study in which an intermediate dose of AAV-mSPB (10^11vg/mouse) was administered with dox removal 7 days later (Fig 6d and Fig 6f, Supplementary Fig 8f). In the initial study (Fig 6d) median survival was 194 days and in the replication study (Fig 6f, Supplementary Fig 8f) median survival was 202 days demonstrating successful replication. We performed 5 survival studies with AAV human SPB using different dosages, with different times of dox removal, and in both neonatal and adult mice. We performed 1 lung structure and function study using AAV murine SPB, and 1 lung structure and function study using AAV human SPB. In all instances the findings were consistent with the AAV-murine SPB and -human SPB groups significantly improving structure/function and survival compared to negative control groups.
Randomization	The Animal Care and Veterinary Service Committee at the University of Ottawa allows 4 mice to be housed per cage, and they are to be grouped with their litter-mates and be of the same sex. During weaning, mice were assigned a numerical ID and uniquely ear-notched by a research technician. The ear-notch corresponded with a particular numerical ID. The survival and structure/function studies all contained both control and treatment groups within a single cage. The treatment of mice (either control or AAV-mSPB/-hSPB) was assigned based on alternating numerical IDs. For example, in a cage of mice with the IDs 1, 2, 3, and 4: 1 and 3 would be administered AAV-SPB, while 2 and 4 would receive a negative control (ie 1×PBS). The order of surgical AAV administration was based on their numerical order: 1st: 1 (AAV-SPB); 2nd: 2 (1×PBS); 3rd: 3 (AAV-SPB); 4th: 4 (1×PBS). For the structure/function analyses using AAV-mSPB or -hSPB (Figs. 4, 5, 7), in a cage of 4 mice 2 were untreated and 2 treated with AAV-SPB in a similar fashion to the survival studies. The mice on dox were carefully chosen for similar dates of birth and sex. A detailed summary including dates of birth and sex for these studies are provided in Supplementary Table 4. For the mouse inflammatory cytokine studies, mice were randomly matched for sex and dates of birth between mice on dox and those treated with AAV-mSPB. In the PCLS studies using human fetal lung, there was no random allocation and covariates were not controlled. The fetal lung samples were chosen if they displayed no underlying anomalies by ultrasound, had no known preexisting medical conditions, and were given consent for research use by the patient after the elective procedure. However, with each human fetal lung sample (n=3 total), negative controls (untransduced PCLS) were always performed.
Blinding	The decision for identifying the respiratory signs and symptoms for euthanasia were made by the staff at ACVS or by members of our group blinded to the identity and treatment groups of the animals. Once the animals were euthanized, we identified which treatment group the mouse belonged to based on their assigned ID (ear notch). In Supplementary Table 5 we provide the information on how many mice were found dead versus euthanized in each survival study. Much of our data was collected and processed by software and so blinding was not required including: IVIS imaging quantification by LivingImage 3.2 software; flow cytometry data collected by BDFACSDiva v8.0; RT-qPCR data processed by StepOnePlus software 3.2; flexiVent data collected by FlexiWare v7.0; and cytokine measurements performed by BDFACSDiva v8.0. However, the histological, immunofluorescence, electron microscope, and confocal images were obtained by operators not blinded to the treatment groups. We did not quantitate any data from these images and provided representative images. We attempted to represent images in a forthcoming manner. For example, we obtained scanned images of the whole lungs and presented as large fields of view of the lungs in the space available. For the IF images we were forthcoming when unexpected SP-B expression was observed (Supplementary Fig 5d), and carried out new experiments to explain these findings (Fig. 5b). For our survival studies we presented all the incidences where untreated mice survived beyond the longest expected survival point of 11 days (Supplementary Fig 6).

Reporting for specific materials, systems and methods

We require information from authors about some types of materials, experimental systems and methods used in many studies. Here, indicate whether each material, system or method listed is relevant to your study. If you are not sure if a list item applies to your research, read the appropriate section before selecting a response.

Materials & experimental systems

n/a	Involved in the study
☐	☒ Antibodies
☐	☒ Eukaryotic cell lines
☒	☐ Palaeontology and archaeology
☐	☒ Animals and other organisms
☐	☒ Human research participants
☒	☐ Clinical data
☒	☐ Dual use research of concern

Methods

n/a	Involved in the study
☒	☐ ChIP-seq
☐	☒ Flow cytometry
☒	☐ MRI-based neuroimaging

Antibodies

Antibodies used

Immunoblotting: We used the c-Myc primary antibody at a 1/2,000 dilution (Cell Signaling; D84C12); Flow Cytometry: The samples were resuspended with the following antibody dilutions: 1/100 CD31-BV421 (Biolegend; 102424); 1/100 CD45-AF647 (Biolegend; 103124); and 1/100 EpCam-PE-CY7 (Biolegend; 118216); Immunofluorescence: Mouse lung tissue samples were incubated with the primary antibodies at a dilution of 1/250 proSP-C (Millipore Sigma; AB3786) or 1/500 SP-B (Seven Hills Bioreagents; WRAB-48604). Human fetal PCLS were stained at a dilution of 1/50 firefly luciferase (Novus; NB100-1677), 1/200 mCherry (Novus; NBP-1-96752), or 1/50 EpCAM (abcam; ab71916). Secondary antibody staining for the western blot was with an anti-rabbit HRP polyclonal secondary antibody at 1/4000 (Invitrogen IgG (H+L); LS656120). Secondary antibody staining was with 1/500 of goat α-rabbit AF-568 (Life technologies; A11011), 1/500 of donkey α-rabbit AF-488 (Invitrogen; A21206), 1/500 of goat α-mouse AF-568 (Invitrogen; A21124), or 1/500 of donkey α-goat AF-568 (Invitrogen; A11057).

Validation

The c-Myc antibody was validated for Western blotting by the manufacturer (cellsignal.com). It was validated for western blotting in multiple cell lines including c-Myc knockout HEK293 cells. Reactivity for human, mouse and rat. Recommended 1:1000 dilution for western blotting. The CD31-BV421, CD45-AF647, and EpCAM-PE-Cy7 antibodies were validated for flow cytometry by the manufacturer (biolegend.com). All 3 antibodies have their lots quality control tested by immunofluorescent staining with flow cytometry analysis. All 3 antibodies are reactive for mouse. The recommended use for CD31-BV421 is 5μL per million cells in 100μL staining volume, and for both CD45-AF646 and EpCAM-PE-Cy7 is ≤0.25 per 10E6 cells in 100μL volume. The proSP-C and SP-B antibodies were validated for immunofluorescence using lung tissues from mice with homozygous Sftpc (Supplementary Fig. 5f) and Sftpb (Supplementary Fig. 5g) gene deletions respectively. The proSP-C antibody was also validated for ELISA, immunohistochemistry (IHC), and western blotting by Millipore Sigma. Reactive for human, mouse and rat. Recommended use for IHC is 1:2000 to 1:4000 in human lung tissue, 1:1000 to 1:2000 in adult and 1:500 to 1:1000 in fetal mouse lung. The SP-B antibody was validated for western blotting, ELISA, and IHC by Seven Hills Bioreagents. Reactive for human, mouse, cow, and sheep. Recommended use for IHC is 1:1000. The Luciferase and mCherry antibodies were validated for immunofluorescence by the manufacturer (novusbio.com). Both antibodies were validated for western blotting, immunofluorescence (IF), and IHC. The luciferase antibody is reactive in human and mouse cell lines and its recommended use for IF is 20μg/mL. The mCherry antibody is reactive in human, mouse, rat, drosophila, and insects and its recommended use for IF is 1:500. The EpCAM antibody was validated for immunofluorescence by the manufacturer (abcam.com). It is validated for western blotting, IF, and IHC. Reactive for mouse, rat, dog, and human. Recommended use for IF is 1 to 5μg/mL.

Eukaryotic cell lines

Policy information about cell lines

Cell line source(s)

HEK293 (ATCC; CRL-1573) and MLE12 (ATCC; CRL-2110)

Authentication

The HEK293 cell line was purchased directly from ATCC and cytogenetic analysis (karyotyping) was performed by ATCC, but further authentication was not performed by our lab. The MLE12 cells were not authenticated.

Mycoplasma contamination

The cell lines were tested and were all negative for mycoplasma contamination.

Commonly misidentified lines
(See ICLAC register)

None used.

Animals and other organisms

Policy information about studies involving animals; ARRIVE guidelines recommended for reporting animal research

Laboratory animals

We used a compound transgenic laboratory mouse (FVB/N strain) that conditionally expresses SP-B under the control of the rtTA protein in AT2 cells. The generation of this mouse is described in detail in Melton KR et al., Am J Physiol Lung Cell Mol Physiol 285: L543-L549 (2003). All attempts were made to use equal numbers of males and females, and use mice between the ages of 6 to 8 weeks at the time of AAV administration. Occasionally we used older mice if we needed larger sample sizes, or younger mice when we performed neonatal survival studies (P8 to P15). For the structure/function and survival studies a total of 276 inducible SP-B mice were used comprising of 124 males, 138 females, and 14 of unknown sex. A detailed summary of the sex and date of birth of each animal used in each experiment in this study is listed in Supplementary Tables 4 and 5. The preceding experiments were performed at the University of Ottawa animal facility where the animals were on a 12 h on, 12 h off light cycle, room temperature was 21C, and humidity was 45%. These are all within the range of guidelines set by the Canadian Council on Animal Care. We also used five male 8-week old Rosa26-Flox/LacZ (Strain: B6.129S4-Gt(ROSA)26Sortm1Sor/J; strain code 003474) mice purchased from Jackson Laboratory (Bar Harbor, ME) for targeting studies to determine the tissues and cell-types transduced by AAV6.2FF. These experiments were

	performed at the University of Guelph and animals were on a 12 h on, 12 h off light cycle, room temperature was 23 to 24C, and humidity was 30-60%.
Wild animals	This study did not involve wild animals.
Field-collected samples	This study did not collect samples from the field.
Ethics oversight	For all animal studies, the study protocol and procedures were approved by the Animal Care and Veterinary Service Committee at The University of Ottawa (Protocol OHRI-1696), and the Animal Care Committee at the University of Guelph (AUP #3827).

Note that full information on the approval of the study protocol must also be provided in the manuscript.

Human research participants

Policy information about studies involving human research participants

Population characteristics	Three human fetal lung tissue samples were used having gestational ages of 16+0 (n=1) and 16+3 (n=2) weeks. The sex, genotype, diagnosis, and treatment categories for all human fetal lung samples were unknown.
Recruitment	Human fetal tissue samples were obtained after elective abortions, and after parental consent from donors. The fetuses contained no anomalies as assessed by ultrasound, and patients with known preexisting medical conditions such as HIV were excluded. The sex of the fetus was unknown. Even if there was a selection bias, there were always some PCLS from each fetus that was used as a negative control (ie untransduced).
Ethics oversight	All human fetal tissues in this study were obtained after receiving institutional review board approval from The Ottawa Hospital (Research Protocol 20170603-01H).

Note that full information on the approval of the study protocol must also be provided in the manuscript.

Flow Cytometry

Plots

Confirm that:

- [x] The axis labels state the marker and fluorochrome used (e.g. CD4-FITC).
- [x] The axis scales are clearly visible. Include numbers along axes only for bottom left plot of group (a 'group' is an analysis of identical markers).
- [x] All plots are contour plots with outliers or pseudocolor plots.
- [x] A numerical value for number of cells or percentage (with statistics) is provided.

Methodology

Sample preparation	Eight days after IT administration of 10^11 vg of either AAV-Luc or AAV-GFP, mice were intraperitoneally injected with heparin, and euthanized. Their thorax region was surgically opened, and the lung vasculature was perfused with 1×PBS (no Ca2+ and Mg2+) and undiluted heparin to remove red blood cells (RBC). Following perfusion, an enzyme mix containing neutral protease, Collagenase I, DNase I in DPBS with Mg2+/Ca2+ was instilled into the lungs. The lungs were removed and placed in the enzyme mix for 1 hr. All traces of the trachea, bronchi, and heart tissue were removed, and then the lung lobes were dissociated into pieces and passed through a cell strainer. The filtered lung suspensions were collected and washed. Cold RBC lysis buffer was added to the cells before the total cell number was counted for each sample. A total of 5 × 10^5 cells were placed into individual wells of a 96-well plate for staining. All samples were blocked with Heavy chain (Fc) block in the dark. The samples were resuspended in FACS buffer with their respective antibodies and incubated for 30 min. The cells were washed, and then resuspended in FACS buffer plus 7-AAD for 5 to 10 min before flow cytometry. No stain, single stain, and fluorescence minus one control stains were all carried out.
Instrument	The samples were analyzed on the BeckmanCoulter MoFlo XDP flow cytometry machine.
Software	Sample compensation and data was analyzed using FlowJo version 10.
Cell population abundance	A total of 6 mice were used with n=2 controls injected with AAV6.2FF-Luciferase, and n=4 treatments injected with AAV6.2FF-GFP. In the 2 control mice, one mouse had 330,022 single viable cells isolated from their lungs with 15,305 CD45-/EpCAM++, and 5 cells CD45-/EpCAM++/GFP+; and the second mouse had 258,617 single viable cells isolated from their lungs with 13,813 CD45-/EpCAM++, and 4 cells CD45-/EpCAM++/GFP+. In the 4 treatment mice, one mouse had 271,917 single viable cells isolated from their lungs with 13,329 CD45-/EpCAM++, and 2,437 cells CD45-/EpCAM++/GFP+; the second mouse had 322,449 single viable cells isolated from their lungs with 35,502 CD45-/EpCAM++, and 11,142 cells CD45-/EpCAM++/GFP+; the third mouse had 194,580 single viable cells isolated from their lungs with 6,176 CD45-/EpCAM++, and 1,024 cells CD45-/EpCAM++/GFP+; and the fourth mouse had 206,691 single viable cells isolated from their lungs with 7,806 CD45-/EpCAM++, and 1,565 cells CD45-/EpCAM++/GFP+. The raw data files for the flow cytometry experiment are provided in Supplementary Table 1 and the Source Data Files.
Gating strategy	The cell populations are represented as contour plots, with outlier events greater than 2% represented as dots. The gating was set as follows: i; Forward Scatter (FSC) vs Side Scatter (SSC) to denote the total cell population from dissociated mouse lungs as represented by size and intracellular granularity respectively. ii; Doublet exclusion to ensure that only single cells are

analyzed by plotting the height against area for SSC. iii; The live (viable) cell population identified as 7AAD negative to ensure that only living cells are analyzed. iv; CD45-AF647 and CD31-BV421 to identify hematopoietic cells (leukocytes) and lung endothelial cells respectively. v; the percentage of CD45-AF647 cells that are GFP+. vi; EpCAM PE/Cy7 and CD31-BV421 to identify lung epithelial and endothelial cells respectively. The EpCAM PE/Cy7 positive cells were divided into lower expressing (dim) and higher expressing (++) EpCAM populations. The EPCAM dim population represents AT1 cells. The EpCAM++ population represents AT2 and ciliated cells. vii; The percentage of GFP+ cells from the total EpCAM PE/Cy7 dim population. viii; The percentage of GFP+ cells from the total EpCAM PE/Cy7++ cell population. ix; The percentage of GFP+ cells from the total CD31-BV421+ cell population. The gating strategy is described in detail in Supplementary Fig. 3.

☒ Tick this box to confirm that a figure exemplifying the gating strategy is provided in the Supplementary Information.
