# Supplementary material for: A lung tropic AAV vector improves survival in a mouse model of surfactant B deficiency
Source: Nat Commun. 2020 Aug 6;11:3929. doi: 10.1038/s41467-020-17577-8 (PMC7414154; doi:10.1038/s41467-020-17577-8)
Supplement: Supplementary file 1 — Supplementary Information [file 41467_2020_17577_MOESM1_ESM.pdf]

Supplementary Figures and Tables

A Lung Tropic AAV Vector Improves Survival in a Mouse Model of Surfactant B Deficiency

Kang MH et al.

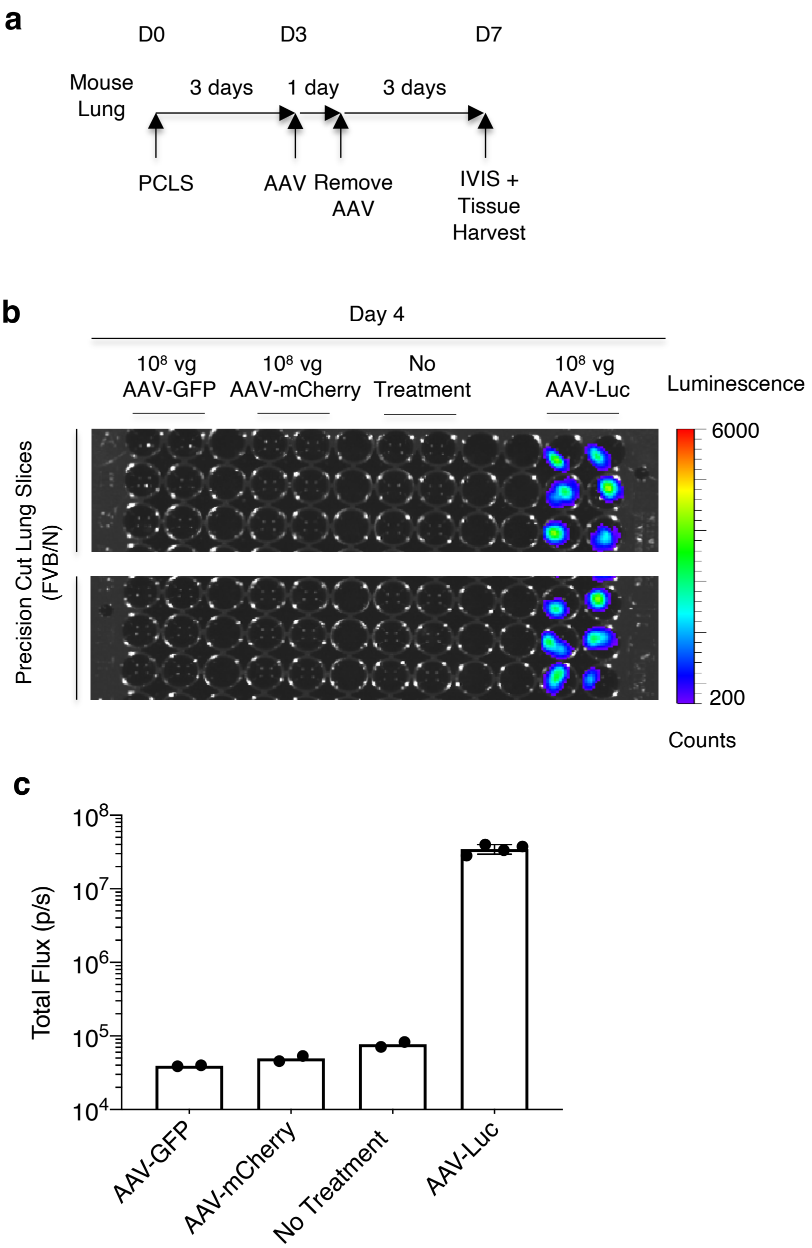

Supplementary Fig. 1 Precision Cut Lung Slices (PCLS) from adult mouse lung tissue (Genetic strain: FVB/N) transduced with AAV6.2FF. a Study design to demonstrate transduction of adult mouse lung tissue slices by AAV6.2FF. Metabolic (resazurin) assays were carried out on days 0, 3, and 7 post-tissue harvest to ensure PCLS metabolic activity and cell viability. **b** IVIS imaging of mouse PCLS 4 days after AAV-Luc transduction and 7 days after generating the tissue slices demonstrates luciferase expression in the adult mouse lung parenchyma following the addition of D-luciferin substrate. The scale is represented as counts. **c** Quantification of bioluminescence from the IVIS image in **(b)** as mean total flux (photons per second) with SD for AAV-Luc group only (All treatment groups contain n=2 animals, 1 lung each with 6 slices per lung, except AAV-Luc with n=4 animals, 1 lung each with 6 slices per lung).

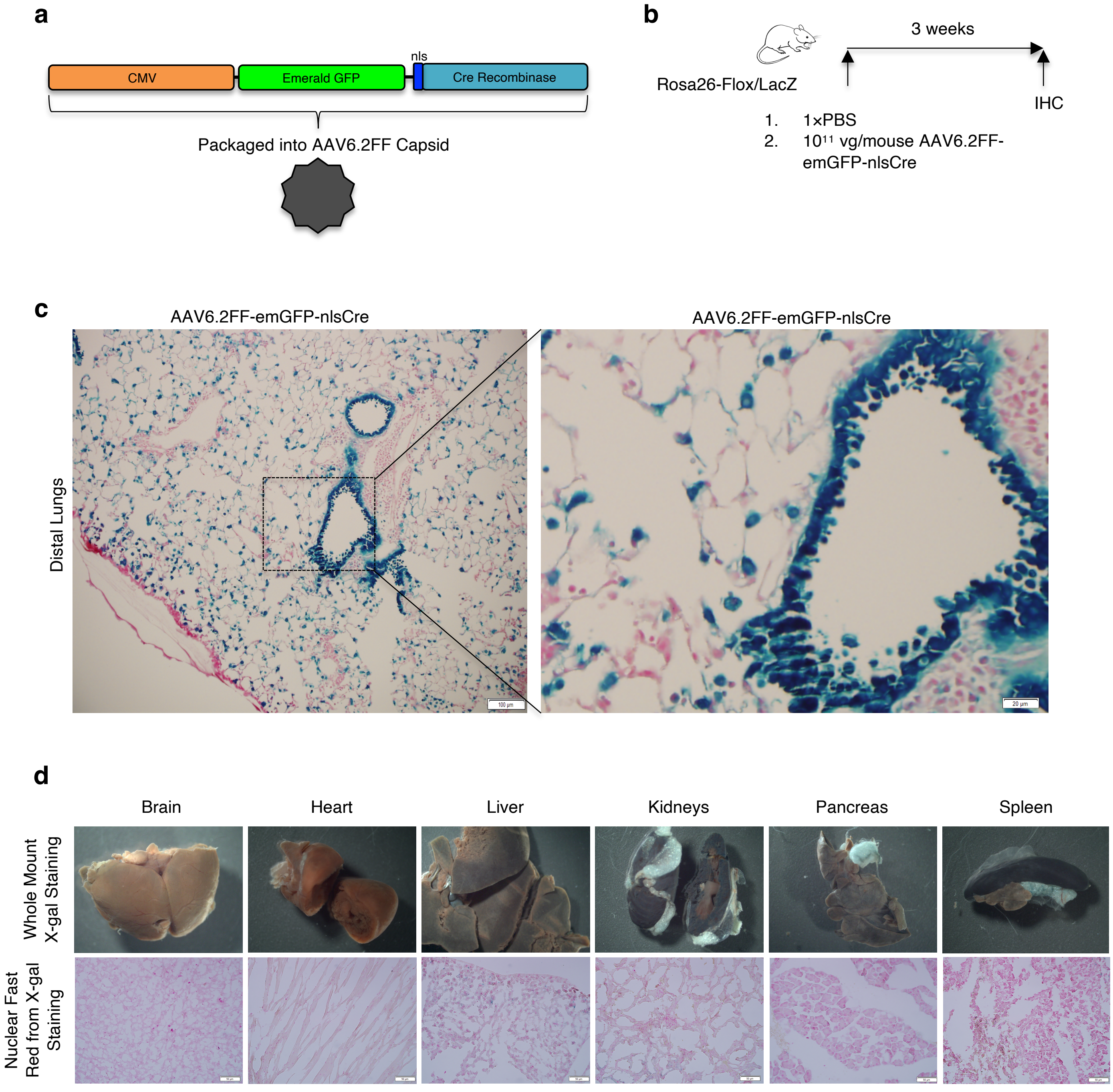
a
CMV
Emerald GFP
nls
Cre Recombinase
Packaged into AAV6.2FF Capsid
b
Rosa26-Flox/LacZ
3 weeks
IHC
1. 1×PBS
2. 10¹¹ vg/mouse AAV6.2FF-emGFP-nlsCre
c
AAV6.2FF-emGFP-nlsCre
AAV6.2FF-emGFP-nlsCre
Distal Lungs
100 µm
20 µm
d
Brain
Heart
Liver
Kidneys
Pancreas
Spleen
Whole Mount X-gal Staining
Nuclear Fast Red from X-gal Staining

Supplementary Fig. 2 Intranasal delivery of AAV6.2FF expressing Cre recombinase mediates recombination in multiple respiratory tract and distal lung cell types but not in other organs. a Schematic of the recombinant AAV2 (rAAV2) vector genome containing a CMV promoter, emerald(em)-GFP, a nuclear localization signal (nls) and Cre recombinase (AAV6.2FF-emGFP-nlsCre) pseudotyped with the AAV6.2FF capsid. **b** Study design to demonstrate the extent of airway and lung cell transduction 3 weeks following modified intranasal (IN) instillation of phosphate-buffered saline (PBS) or 10^{11} vector genomes (vg) of AAV6.2FF-emGFP-nlsCre into 6-week-old Rosa26-Flox/LacZ (B6.129S4-Gt(ROSA)26Sor^{tm1Sor}/J) mice. **c** AAV6.2FF expressing Cre mediates functional recombination in multiple cell types in the mouse distal lung. 6-week-old Rosa26-Flox/LacZ (B6.129S4-Gt(ROSA)26Sor^{tm1Sor}/J) mice were administered 10^{11} vector genomes (vg) of AAV6.2FF-CMV-emGFP-nlsCre using a modified intranasal instillation method. Representative nuclear fast red counterstained paraffin sections (4 mm) from X-gal stained lungs shows abundant LacZ staining in the lung parenchyma, especially of distal airway and alveolar epithelial cells in the magnified image as indicated by the black box (Scale bar, 100 µm; Magnified image scale bar, 20 µm). This image displays a different field of view from the same image as in Fig. 2b (Distal Lungs from a AAV6.2FF-emGFP-nlsCre mouse) with a focus on the distal conducting airways. **d** AAV6.2FF-emGFP-nlsCre does not mediate functional recombination in multiple non-lung tissues including the brain, heart, liver, kidneys, pancreas, and spleen following IN administration (Scale bar, 50 µm).

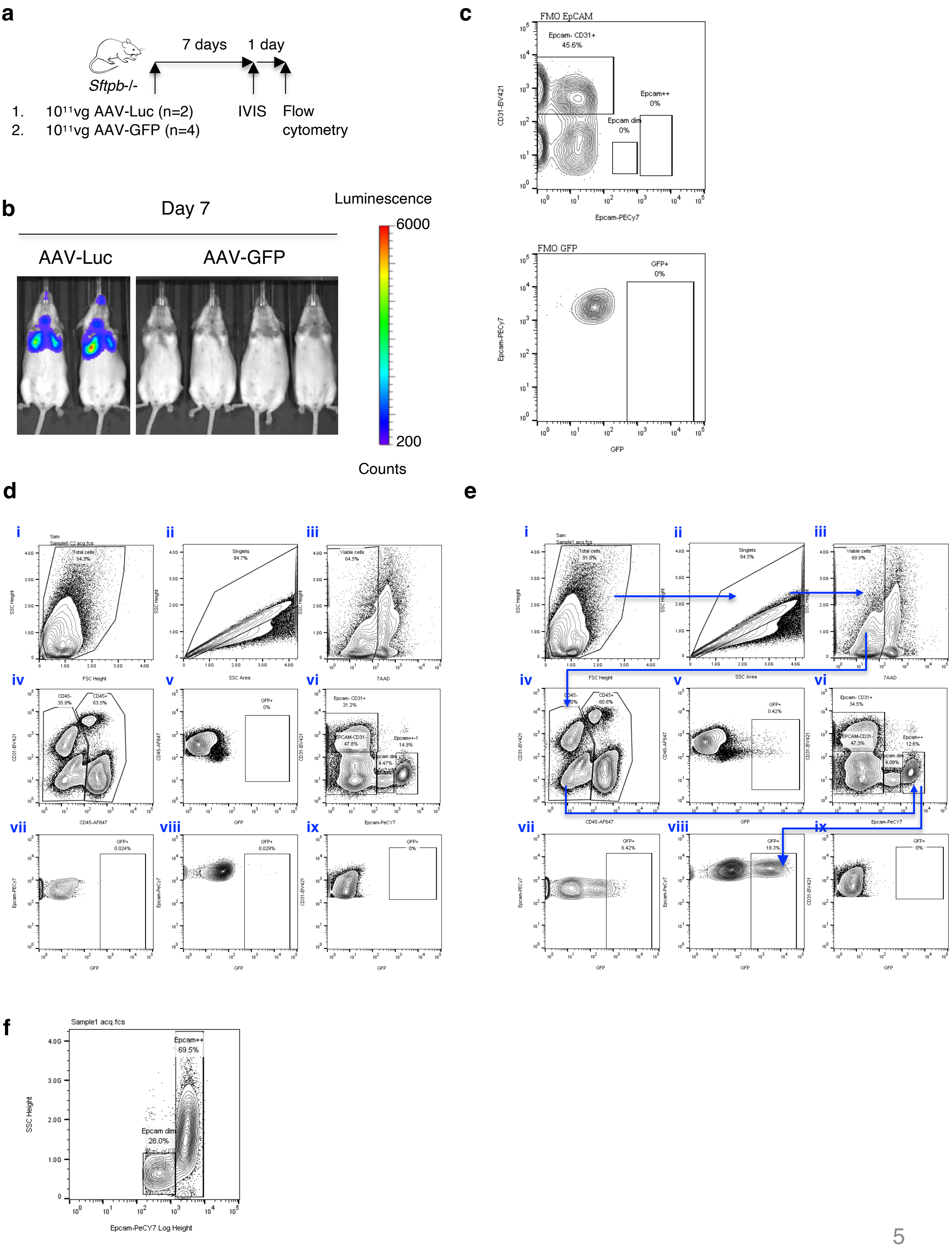
a
7 days
1 day
Sftpb-/-
1. 10¹¹vg AAV-Luc (n=2)
2. 10¹¹vg AAV-GFP (n=4)
IVIS
Flow cytometry
b
Day 7
AAV-Luc
AAV-GFP
Luminescence
6000
200
Counts
c
FMO EpCAM
Epcam- CD31+ 45.6%
Epcam++ 0%
Epcam dim 0%
CD31-BV421
Epcam-PECy7
FMO GFP
GFP+ 0%
Epcam-PECy7
GFP
d
i
Total cells 94.3%
SSC Height
FSC Height
ii
Singlets 84.7%
SSC Area
iii
Viable cells 64.5%
7AAD
iv
CD45- 35.9%
CD45+ 63.5%
CD31-BV421
CD45-AF647
v
GFP+ 0%
vi
Epcam- CD31+ 31.2%
EPCAM-CD31- 47.8%
Epcam++ 14.9%
Epcam dim 4.47%
vii
GFP+ 0.024%
viii
GFP+ 0.029%
ix
GFP+ 0%
e
i
Total cells 91.8%
ii
Singlets 84.5%
iii
Viable cells 69.9%
iv
CD45-
CD45+ 60.6%
v
GFP+ 0.42%
vi
Epcam- CD31+ 34.5%
EPCAM-CD31- 47.3%
Epcam++ 12.6%
Epcam dim 4.08%
vii
GFP+ 6.42%
viii
GFP+ 18.3%
ix
GFP+ 0%
f
Sample1 acq.fcs
Epcam++ 69.5%
Epcam dim 28.0%
SSC Height
Epcam-PeCY7 Log Height

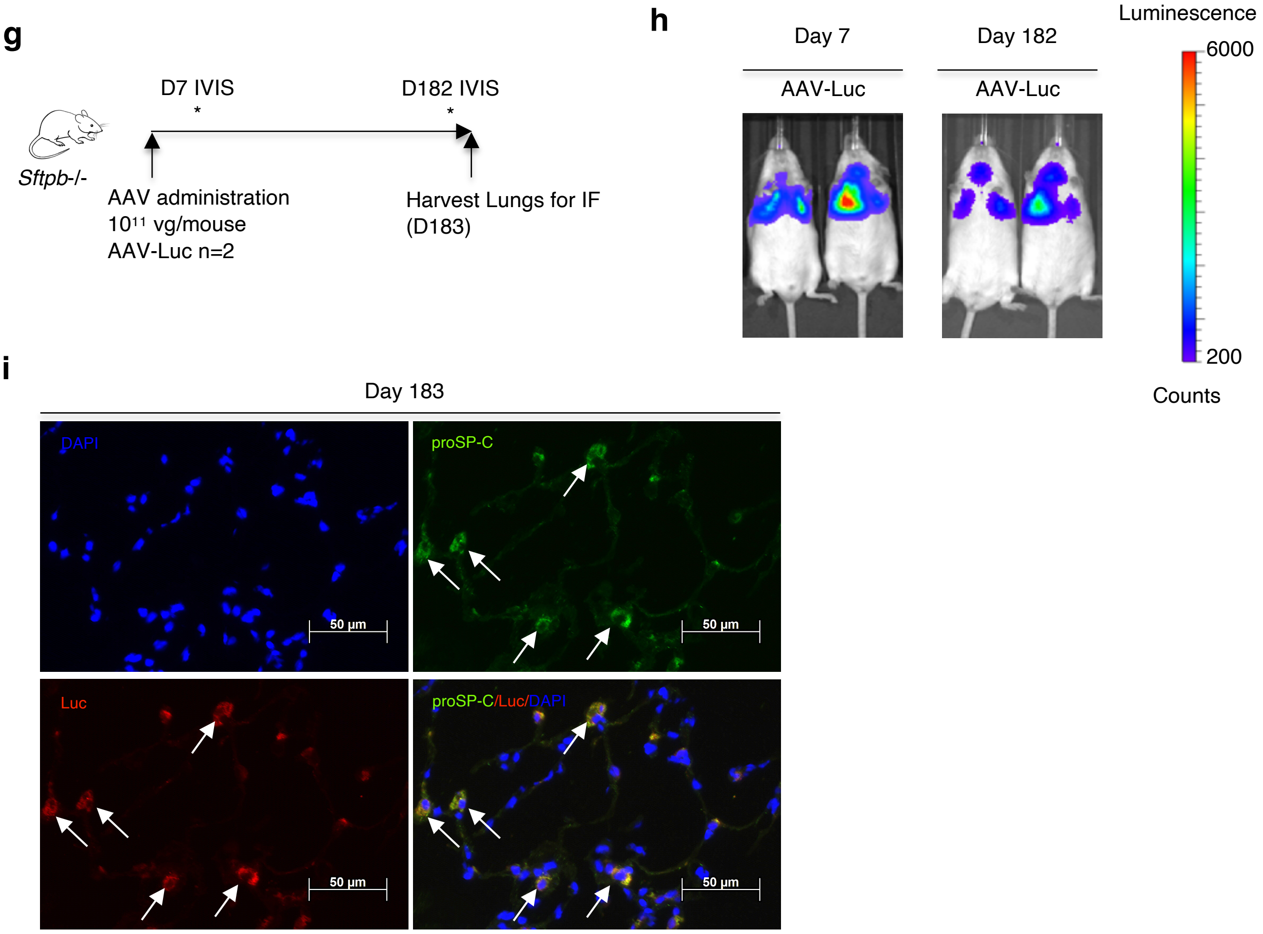
g
D7 IVIS
D182 IVIS
Sftpb-/-
AAV administration
10¹¹ vg/mouse
AAV-Luc n=2
Harvest Lungs for IF
(D183)
h
Day 7
Day 182
AAV-Luc
AAV-Luc
Luminescence
6000
200
Counts
i
Day 183
DAPI
proSP-C
Luc
proSP-C/Luc/DAPI
50 µm

Supplementary Fig. 3 Flow cytometry analysis of dissociated whole mouse lungs from transgenic SP-B mice 8 days following intratracheal administration of 10^{11} vg per mouse of AAV6.2FF-GFP (AAV-GFP). **a** Study design to determine the lung cell types transduced by AAV6.2FF. **b** IVIS imaging 7 days post-administration demonstrates bioluminescence from the AAV6.2FF-Luciferase (AAV-Luc) but not the AAV-GFP animals as expected. **c** Fluorescence minus one (FMO) controls were first carried out to determine the gate settings. The top contour plot contains cells from the lung stained with all the fluorophores minus EpCAM PE/Cy7 to identify the EpCAM cell populations (both low [dim] and high [++] expression). The bottom contour plot represents cells from the lung of a mouse administered with AAV-Luc to identify the GFP negative population. **d** Sample analysis of lungs from a control mouse (AAV-Luc; Sample6 C2). The cell populations are represented as contour plots, with outlier events greater than 2% represented as individual dots. The gating was set as follows: i; Forward Scatter (FSC)/Side Scatter (SSC) to denote the total cell population from dissociated mouse lungs as represented by size and intracellular granularity, respectively. ii; Doublet exclusion to ensure that only single cells are analyzed by plotting the height against area for SSC. iii; The live (viable) cell population identified as 7AAD negative to ensure that only living cells are analyzed. iv; CD45-AF647 and CD31-BV421 to identify hematopoietic cells (leukocytes) and lung endothelial cells, respectively. v; the percentage of CD45-AF647 cells that are GFP positive. vi; EpCAM PE/Cy7 and CD31-BV421 to identify lung epithelial and endothelial cells, respectively. The EpCAM PE/Cy7 positive cells were divided into low (dim) and highly expressing (++) EpCAM populations. The EPCAM dim population represents AT1 cells. The EpCAM++ population represents AT2 and ciliated cells. vii; The percentage of GFP positive cells from the total EpCAM PE/Cy7 dim population. viii; The percentage of GFP positive cells from the total EpCAM PE/Cy7++ cell population. ix; The percentage of GFP positive cells from the total CD31-BV421+ cell population. **e** Sample analysis of lungs from an AAV-GFP mouse (Sample1). The gating strategy is identical to the scheme described for the control mouse in **(d)** above. The gating strategy used to identify CD45-, EpCAM++ (AT2 and ciliated cells) that are GFP+ is indicated by the blue arrows. Gating panel viii corresponds to the flow cytometry data panels in Fig. 2c. **f** A plot of SSC that distinguishes the two EpCAM populations based on intracellular granularity. The EpCAM++ (AT2 and ciliated cells) population demonstrates higher intracellular granularity than the EpCAM dim (AT1 cells) population, likely due to the presence of the lysosomal-like lamellar bodies (LB) in AT2 cells which are not found in AT1 cells. **g** Study design to visually confirm transduction of AT2 cells by AAV-Luc using immunofluorescence imaging. **h** IVIS imaging demonstrates rapid (7 days) and long-term (182 days) AAV expression of firefly luciferase (Luc). **i** Epifluorescence imaging of Luc (green) and proSP-C (red) co-staining 183 days post AAV administration in AT2 cells. Staining for proSP-C, Luc, and their co-localization with DAPI are indicated by the white arrows (Scale bar, 50 µm).

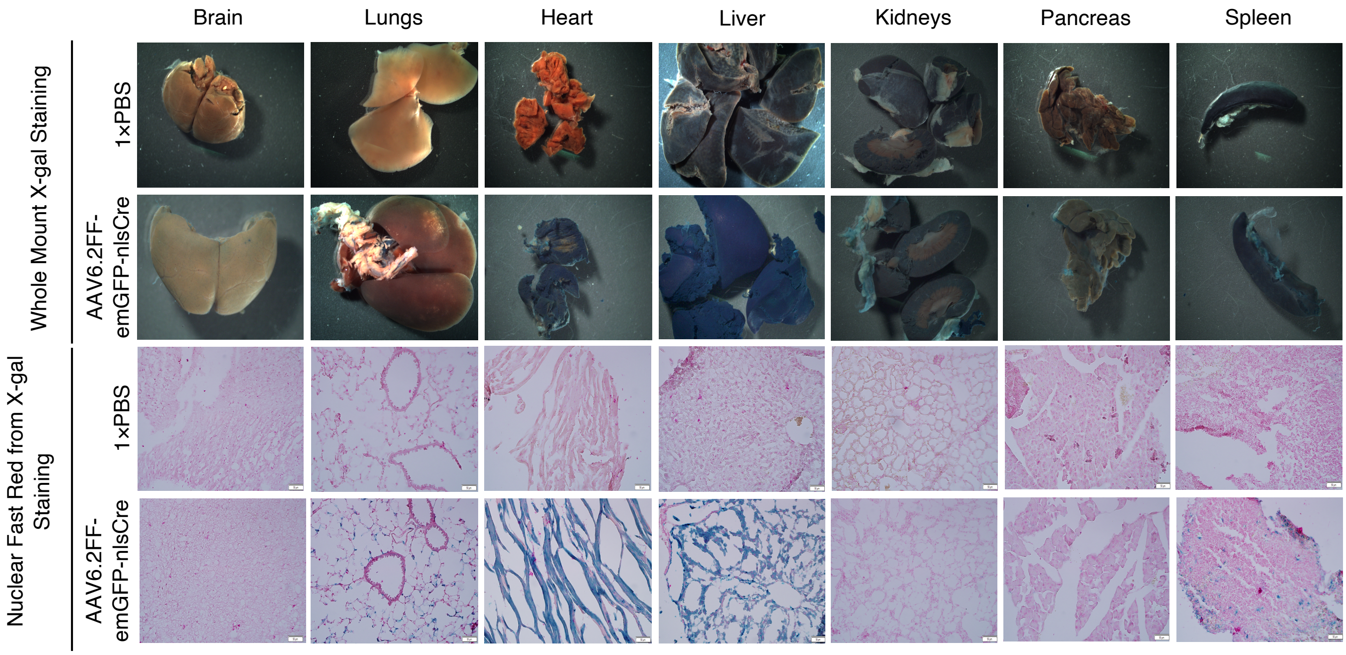

Supplementary Fig. 4 Intravenous administration of AAV6.2FF-emGFP-nlsCre mediates functional recombination in the lungs, heart, liver and spleen, but not in the brain, kidneys or pancreas. Six-week-old Rosa26-Flox/LacZ (B6.129S4-Gt(ROSA)26Sor^{tm1Sor}/J) mice were administered 1×PBS or 10^{11} vector genomes (vg) of AAV6.2FF-CMV-emGFP-nlsCre intravenously via tail vein injection. X-gal staining of tissues was performed 3 weeks post-vector administration, and both representative whole mount gross organ images, as well as nuclear fast red counterstained cryosections (8 µm) from X-gal stained tissues are displayed here (Scale bar, 50 µm).

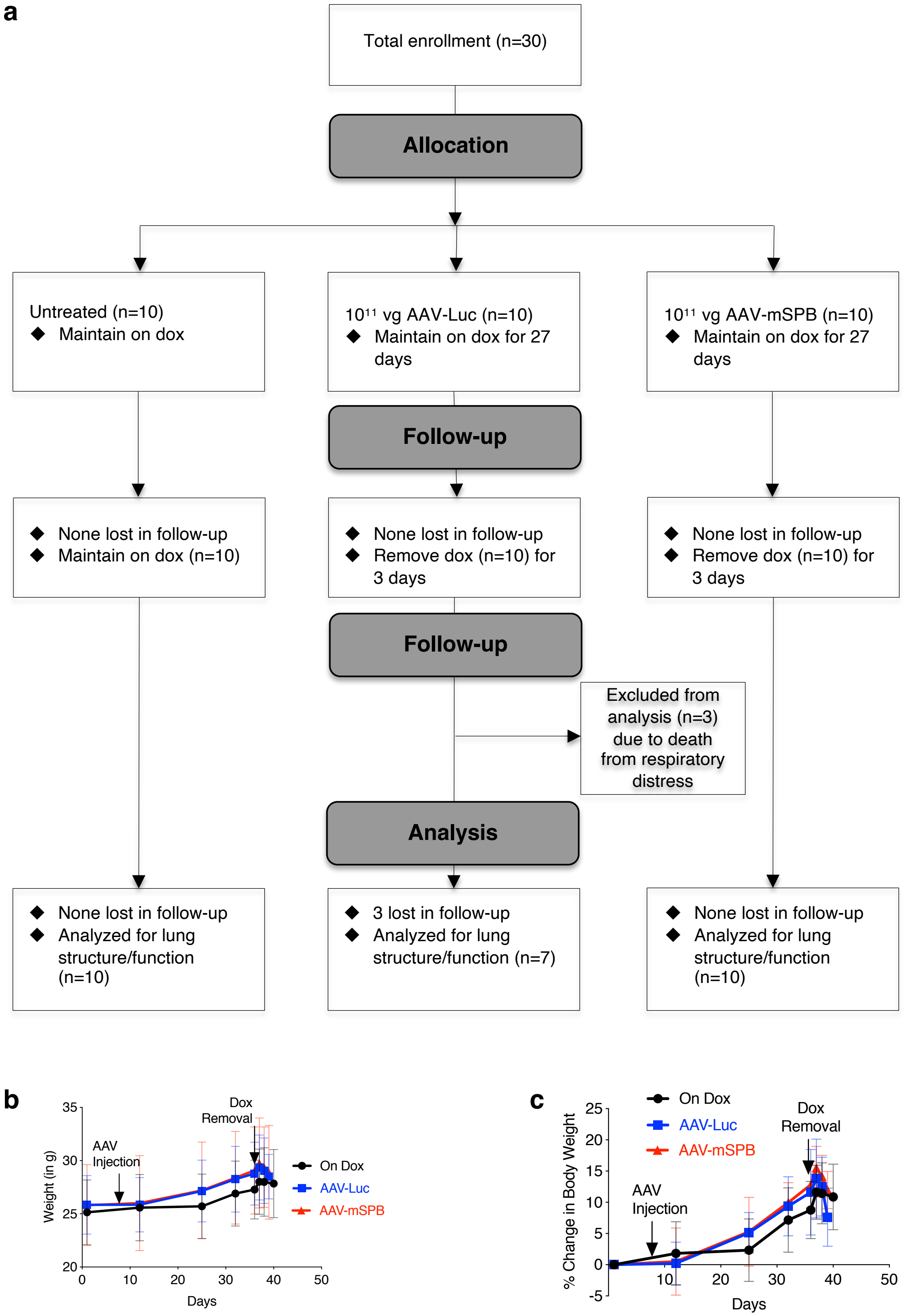
a
Total enrollment (n=30)
Allocation
Untreated (n=10)
Maintain on dox
10¹¹ vg AAV-Luc (n=10)
Maintain on dox for 27 days
10¹¹ vg AAV-mSPB (n=10)
Maintain on dox for 27 days
Follow-up
None lost in follow-up
Maintain on dox (n=10)
None lost in follow-up
Remove dox (n=10) for 3 days
None lost in follow-up
Remove dox (n=10) for 3 days
Follow-up
Excluded from analysis (n=3) due to death from respiratory distress
Analysis
None lost in follow-up
Analyzed for lung structure/function (n=10)
3 lost in follow-up
Analyzed for lung structure/function (n=7)
None lost in follow-up
Analyzed for lung structure/function (n=10)
b
Weight (in g)
AAV Injection
Dox Removal
Days
On Dox
AAV-Luc
AAV-mSPB
c
% Change in Body Weight
On Dox
AAV-Luc
AAV-mSPB
Dox Removal
AAV Injection
Days

d

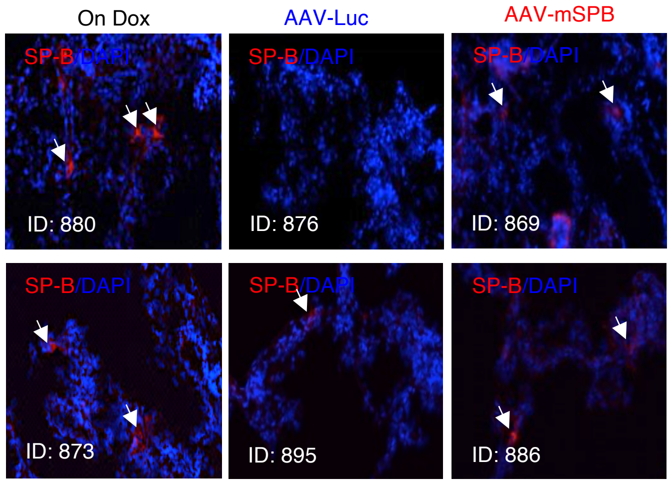
On Dox
AAV-Luc
AAV-mSPB
SP-B/DAPI
ID: 880
ID: 876
ID: 869
ID: 873
ID: 895
ID: 886

e

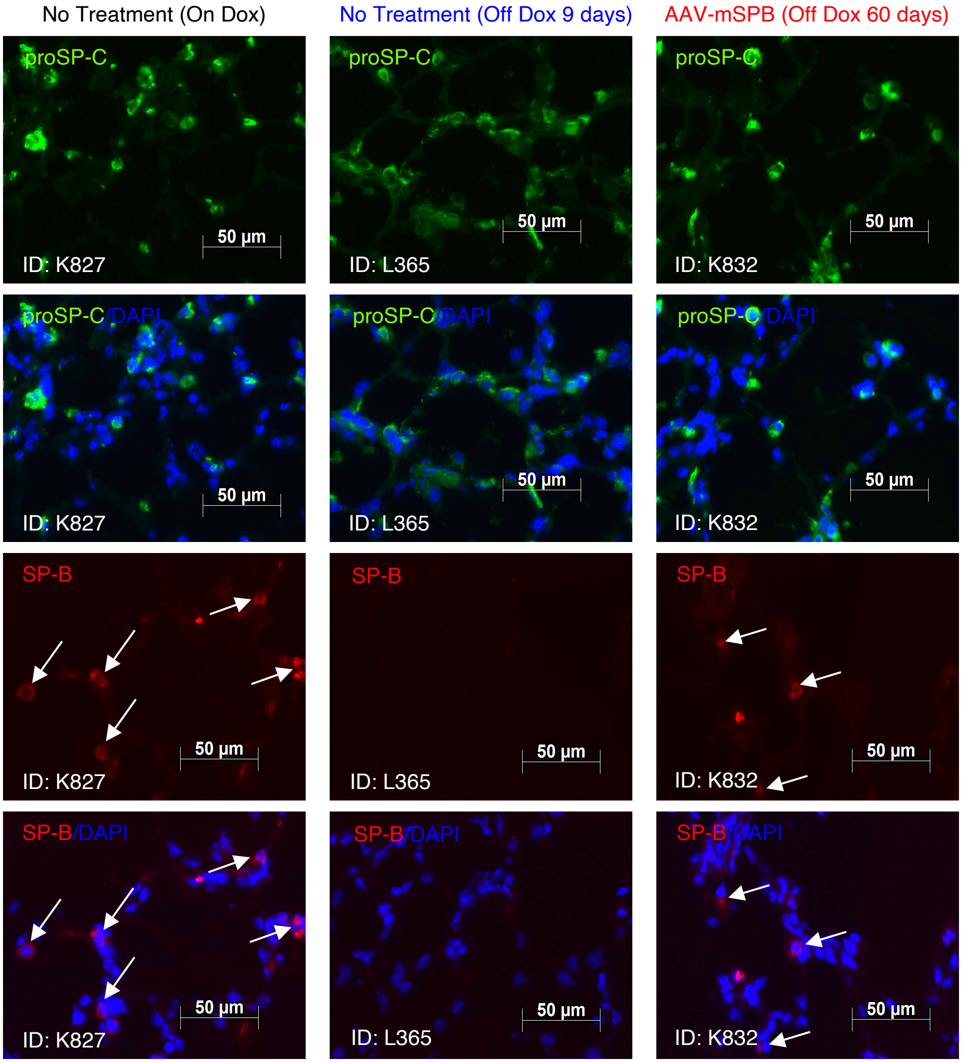
No Treatment (On Dox)
No Treatment (Off Dox 9 days)
AAV-mSPB (Off Dox 60 days)
proSP-C
proSP-C/DAPI
SP-B
SP-B/DAPI
50 µm
ID: K827
ID: L365
ID: K832

f

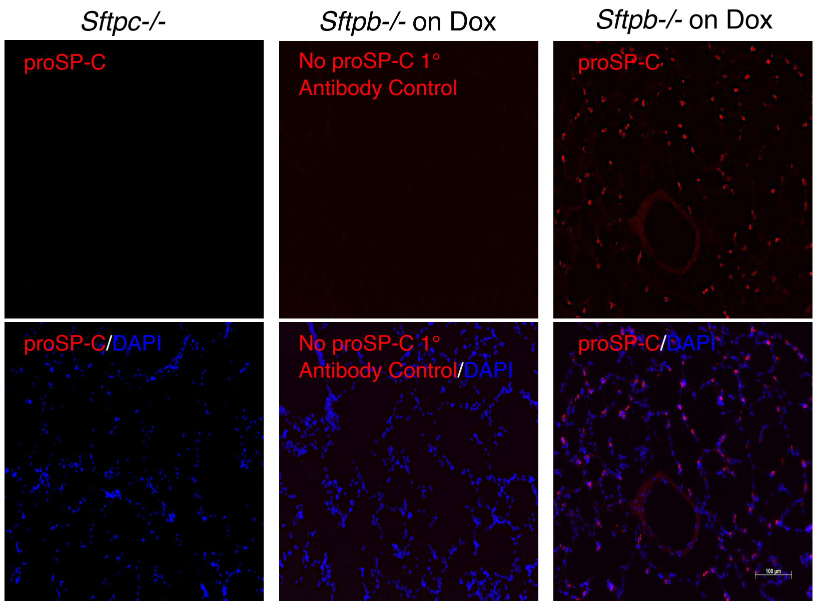

Sftpc-/-
Sftpb-/- on Dox
Sftpb-/- on Dox
proSP-C
No proSP-C 1° Antibody Control
proSP-C
proSP-C/DAPI
No proSP-C 1° Antibody Control/DAPI
proSP-C/DAPI

g

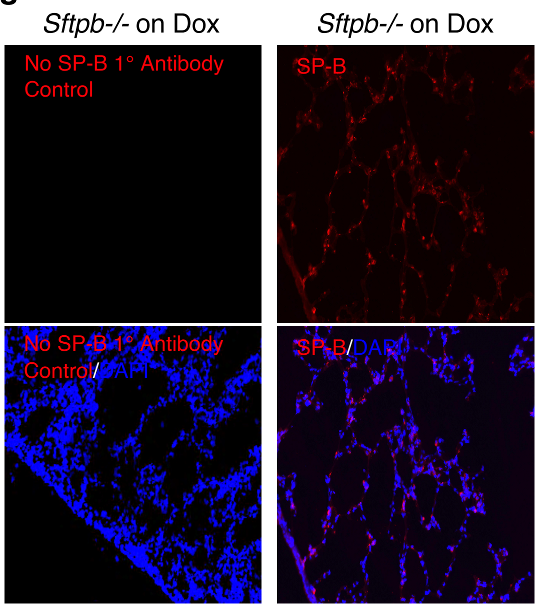

Sftpb-/- on Dox
Sftpb-/- on Dox
No SP-B 1° Antibody Control
SP-B
SP-B/DAPI

h

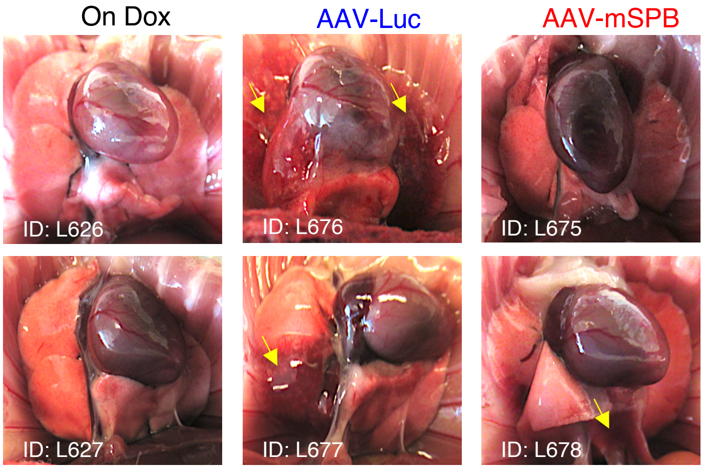

On Dox
AAV-Luc
AAV-mSPB
ID: L626
ID: L676
ID: L675
ID: L627
ID: L677
ID: L678

i.

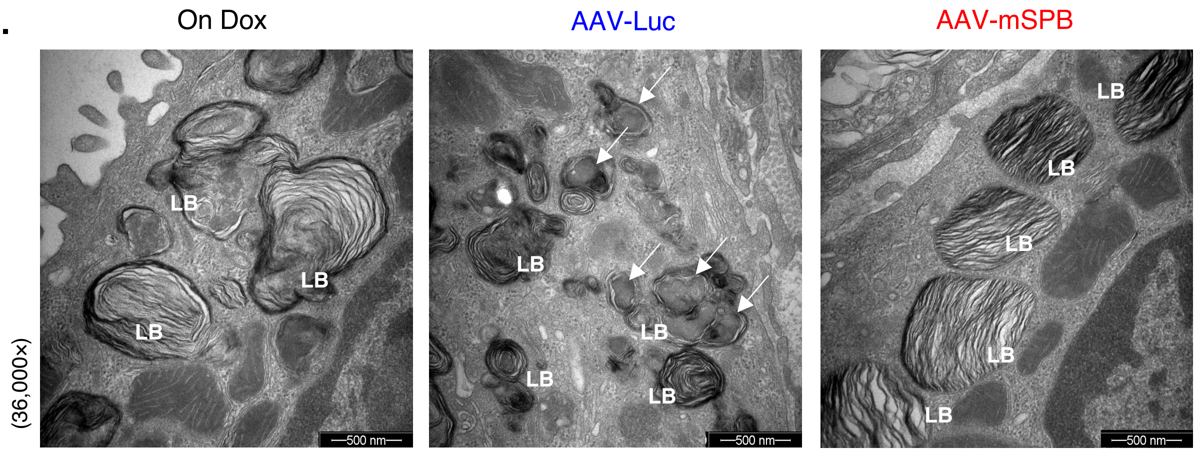

On Dox
AAV-Luc
AAV-mSPB
(36,000×)
LB
500 nm

Supplementary Fig. 5 Structure and function analysis of lungs from SP-B deficient mice intratracheally administered 10^{11} vg per mouse of AAV6.2FF murine *proSftpb* cDNA (AAV-mSPB). a Consort diagram of the AAV-mSPB structure/function study from Fig. 4. **b** Mean weight measurements with SD from all surviving mice in Fig. 4. **c** Percent change in mean body weight with SD from all surviving mice in Fig. 4 (For both **(b)** and **(c)**, n=10 per group at all timepoints until Dox removal where AAV-Luc n=7). **d** Representative epifluorescence images of SP-B (red), and DAPI (blue) from frozen right lung sections 3 days after dox removal. White arrows indicate SP-B staining. **e** Representative epifluorescence images of proSP-C (green), SP-B (red), and DAPI (blue) from frozen lung sections of mice on dox; untreated mice off dox for 9 days; and 10^{11} vg AAV-mSPB treated mice off dox for 60 days (Scale bar, 50 µm). **f** ProSP-C (red) and DAPI (blue) staining controls in a mouse homozygous for *Sftpc* mutations, as well as the inducible SP-B mouse model on dox (Scale bar, 100 µm). **g** SP-B (red) and DAPI (blue) staining controls in the inducible SP-B mouse model on dox. Scale bar in **(f)** applies to this figure. **h** Gross lung images from SP-B deficient mice used in the transmission electron microscopy (TEM) imaging 4 days following dox removal from Fig. 5c. Yellow arrows indicate regions of lung damage. **i** Representative TEM images of AT2 cells 4 days following dox removal. White arrows indicate the accumulation of amorphous material within lamellar bodies (LB) from an AAV-Luc mouse (Scale bar, 500 nm).

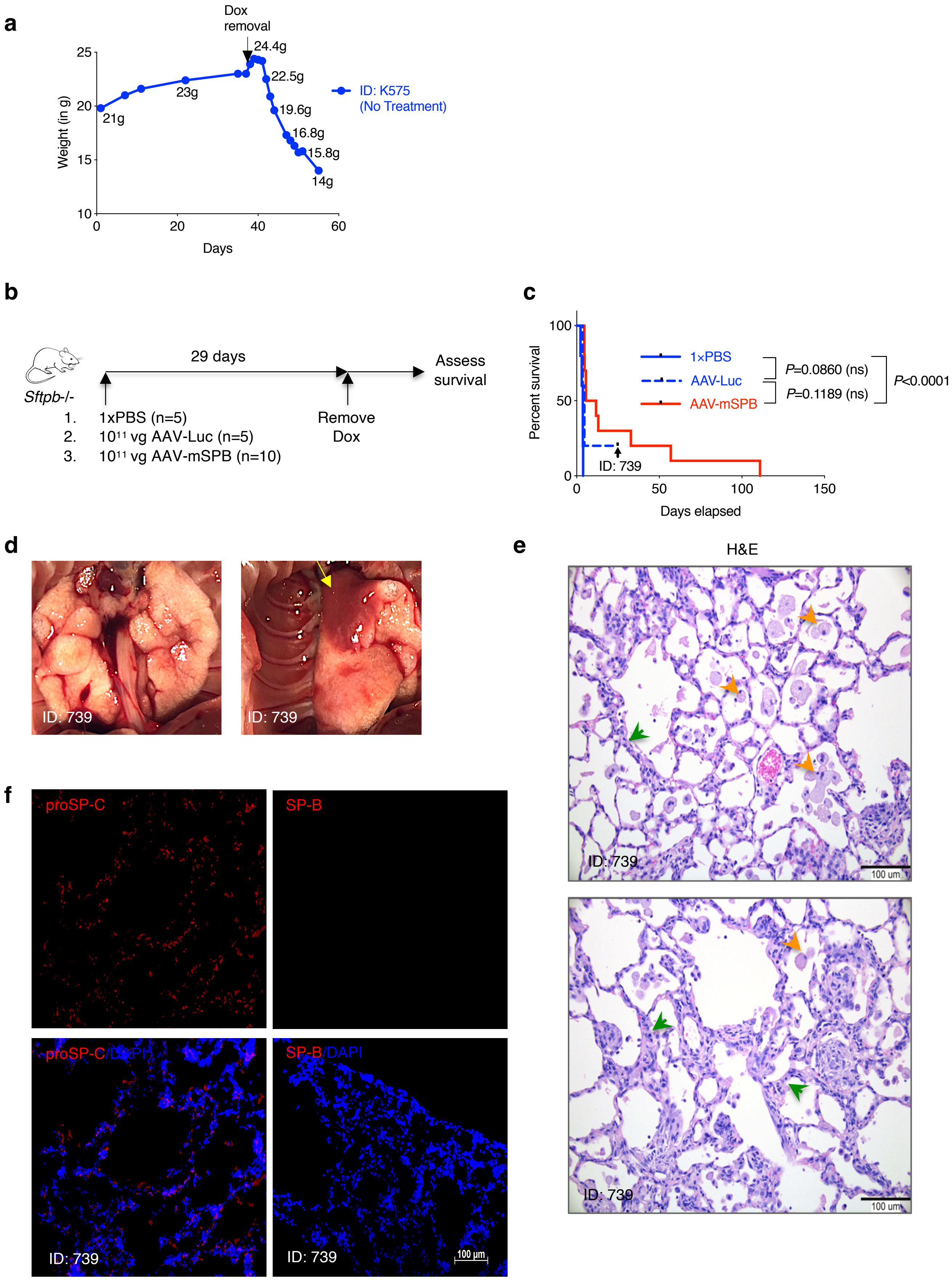
a
Dox removal
24.4g
22.5g
23g
21g
19.6g
16.8g
15.8g
14g
ID: K575 (No Treatment)
Weight (in g)
Days
b
29 days
Assess survival
Sftpb-/-
1. 1xPBS (n=5)
2. 10¹¹ vg AAV-Luc (n=5)
3. 10¹¹ vg AAV-mSPB (n=10)
Remove Dox
c
Percent survival
1×PBS
AAV-Luc
AAV-mSPB
P=0.0860 (ns)
P=0.1189 (ns)
P<0.0001
ID: 739
Days elapsed
d
ID: 739
ID: 739
e
H&E
ID: 739
100 um
ID: 739
100 um
f
proSP-C
SP-B
proSP-C/DAPI
SP-B/DAPI
ID: 739
ID: 739
100 µm

Supplementary Fig. 6 Incidences of untreated or negative control SP-B deficient mice surviving beyond the longest expected endpoint of 11 days off dox. a Body weight of a single untreated mouse from Fig. 6b (ID: K575; n=1) that survived for 18 days off dox. This mouse demonstrated a rapid and significant weight loss of more than 40% following dox removal and was eventually found dead. **b** Study design to assess survival following an intermediate (10^{11} vg per mouse) dose of AAV-mSPB in SP-B deficient mice. Negative control mice underwent sham surgeries with intratracheal (IT) injections of either 1×PBS or 10^{11} vg per mouse AAV-Luc. **c** Kaplan-Meier survival curve of **(b)**. (Survival curve *P* values = 2-tailed Log-rank, Mantel-Cox test with Bonferroni correction, significance is set at P=0.0167; ns = not significant). **d** Lung images from an AAV-Luc treated mouse (ID: 739) 24.83 days following dox removal from the study in **(c)**. The second image of the dorsal right lung indicates a region of discoloration (yellow arrow). **e** Two representative H&E images from paraffin embedded whole left lungs after euthanasia of mouse 739. Green arrows indicates septal wall thickening, and orange arrows indicates cellular infiltration in alveolar spaces (Scale bar, 100 µm). **f** Representative epifluorescence images of proSP-C (red), SP-B (red), and DAPI (blue) staining in frozen right lung sections from mouse 739 demonstrating the absence of SP-B expression (Scale bar, 100 µm).

a

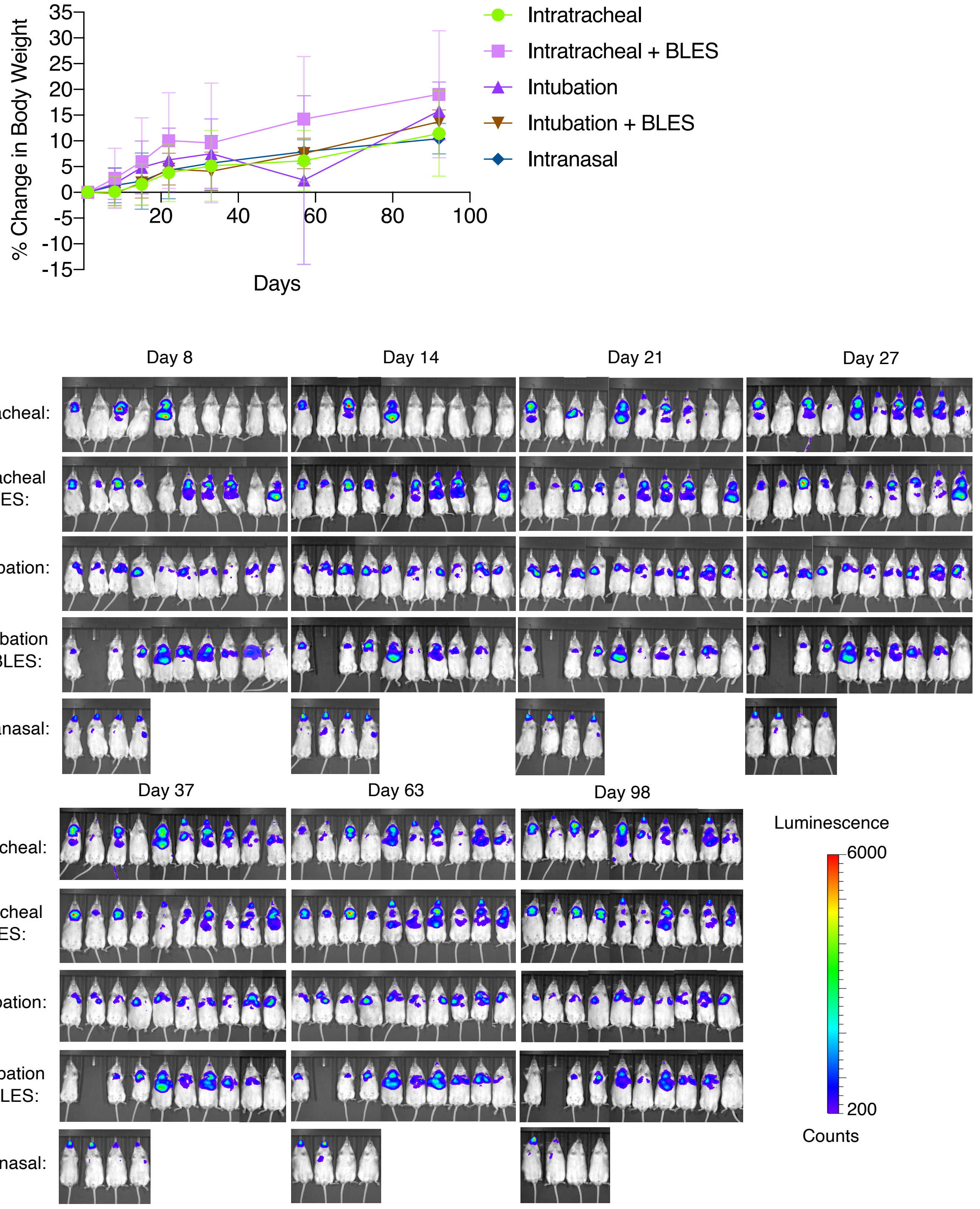

% Change in Body Weight
35
30
25
20
15
10
5
0
-5
-10
-15
20
40
60
80
100
Days
Intratracheal
Intratracheal + BLES
Intubation
Intubation + BLES
Intranasal
b
Day 8
Day 14
Day 21
Day 27
Day 37
Day 63
Day 98
Intratracheal:
Intratracheal + BLES:
Intubation:
Intubation + BLES:
Intranasal:
Luminescence
6000
200
Counts

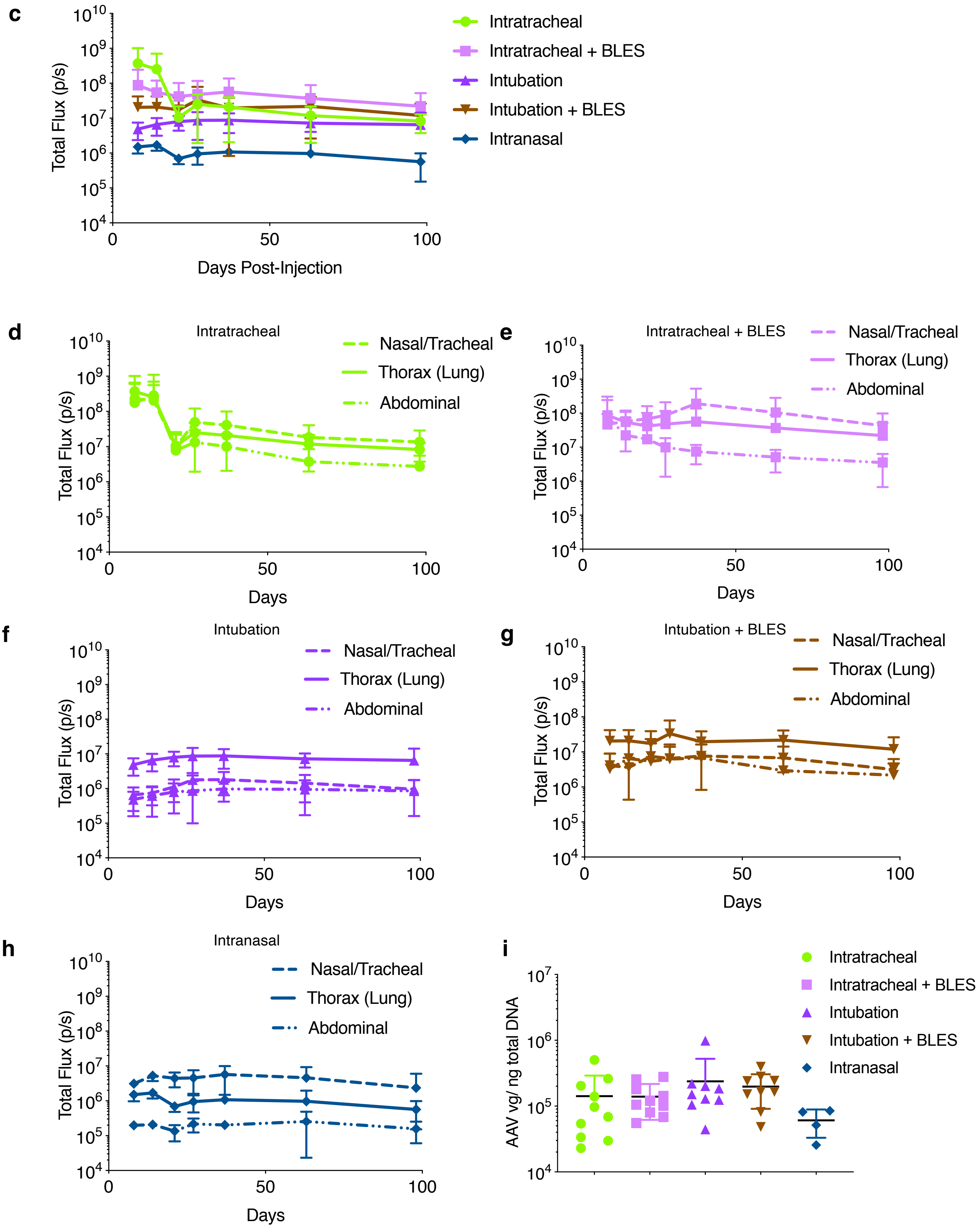
c
Total Flux (p/s)
Days Post-Injection
Intratracheal
Intratracheal + BLES
Intubation
Intubation + BLES
Intranasal
d
Intratracheal
e
Intratracheal + BLES
f
Intubation
g
Intubation + BLES
h
Intranasal
Nasal/Tracheal
Thorax (Lung)
Abdominal
Days
i
AAV vg/ ng total DNA

Supplementary Fig. 7 IVIS imaging of various administration methods into the distal lung including intubation and the use of pulmonary surfactant (Bovine Lipid Extract Surfactant; BLES) as a vehicle for the AAV vector. a Percent change in mean body weight with SD in all mice demonstrates no significant differences following vector delivery between the different administration methods. **b** IVIS imaging of SP-B deficient mice on dox at 8, 14, 21, 27, 37, 63 and 98 days post-injection of 10^{11} vg per mouse of AAV8-Luc by IT (green circle, n=10), IT plus BLES (light purple square, n=10), intubation (dark purple triangle, n=10), intubation plus BLES (brown upside-down triangle, n=9), and intranasal (blue diamond, n=4) administration. **c** Mean bioluminescence quantification as total flux (photons per second) with SD in all groups from the thorax (lung) region. **d** Mean bioluminescence quantification with SD from IT injected mice in the nasal/tracheal (dashed line), thorax (lung) (solid line), and abdominal (solid and dotted line) regions. **e** Mean bioluminescence quantification with SD from IT plus BLES injected mice in the nasal/tracheal, thorax (lung), and abdominal regions. **f** Mean bioluminescence quantification with SD from intubation injected mice in the nasal/tracheal, thorax (lung), and abdominal regions. **g** Mean bioluminescence quantification with SD from intubation plus BLES injected mice in the nasal/tracheal, thorax (lung), and abdominal regions. **h** Mean bioluminescence quantification with SD from intranasal injected mice in the nasal/tracheal, thorax (lung), and abdominal regions (For **(a)** and **(c-h)**, n=10 for IT, IT plus BLES, and intubation; n=9 for intubation plus BLES; and n=4 for intranasal and represents biologically independent animals at all timepoints). **i** qPCR quantification in lung tissue of AAV8 as indicated by mean AAV vg per total DNA in nanograms between the different administration groups at day 99 with SD. The difference in AAV expression in the lungs between the groups was not statistically significant at day 99 by ordinary one-way ANOVA with a Tukey's multiple comparisons post hoc test (For **(i)**, n=10 for IT, and IT plus BLES; n=9 for intubation, and intubation plus BLES; and n=4 for intranasal and represents biologically independent animals at all timepoints).

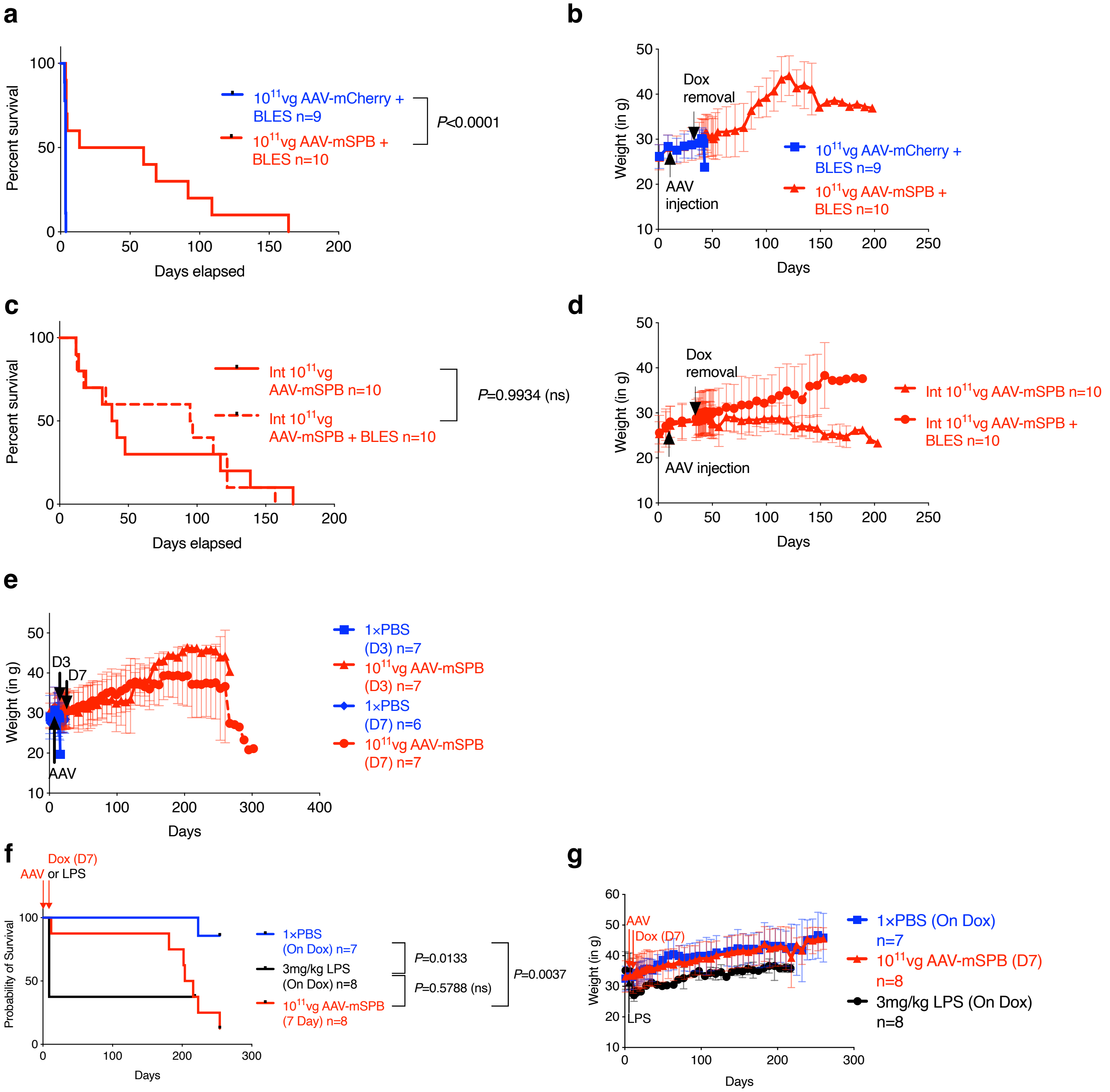
a
Percent survival
Days elapsed
10^11vg AAV-mCherry + BLES n=9
10^11vg AAV-mSPB + BLES n=10
P<0.0001
b
Weight (in g)
Days
Dox removal
AAV injection
10^11vg AAV-mCherry + BLES n=9
10^11vg AAV-mSPB + BLES n=10
c
Percent survival
Days elapsed
Int 10^11vg AAV-mSPB n=10
Int 10^11vg AAV-mSPB + BLES n=10
P=0.9934 (ns)
d
Weight (in g)
Days
Dox removal
AAV injection
Int 10^11vg AAV-mSPB n=10
Int 10^11vg AAV-mSPB + BLES n=10
e
Weight (in g)
Days
D3
D7
AAV
1×PBS (D3) n=7
10^11vg AAV-mSPB (D3) n=7
1×PBS (D7) n=6
10^11vg AAV-mSPB (D7) n=7
f
Dox (D7)
AAV or LPS
Probability of Survival
Days
1×PBS (On Dox) n=7
3mg/kg LPS (On Dox) n=8
10^11vg AAV-mSPB (7 Day) n=8
P=0.0133
P=0.5788 (ns)
P=0.0037
g
Weight (in g)
Days
AAV
Dox (D7)
LPS
1×PBS (On Dox) n=7
10^11vg AAV-mSPB (D7) n=8
3mg/kg LPS (On Dox) n=8

Supplementary Fig. 8 The effects of different administration methods and early dox removal on body weight and median survival in SP-B deficient mice treated with 10^{11} vg per mouse of AAV-mSPB. a Kaplan-Meier survival curve following IT administration of an intermediate (10^{11} vg per mouse) dose of AAV-mSPB plus BLES. **b** Mean weight measurements with SD of all surviving mice demonstrates rapid weight loss before suffering lethal respiratory distress in the control mice compared to a consistent weight gain in the AAV-mSPB treated mice following dox removal. **c** Kaplan-Meier survival curve from intubation (Int) delivery of an intermediate (10^{11} vg per mouse) dose of AAV-mSPB with or without BLES. **d** Mean weight measurements with SD of all surviving mice from **(c)** demonstrate steady weight gains, especially in the group intubated with AAV-mSPB with BLES. **e** Mean weight measurements with SD of all surviving mice from Fig. 6d (IT administration of an intermediate dose of AAV-mSPB with early day 3 (D3) and day 7 (D7) dox removal) demonstrate consistent weight gains for more than 150 days following dox removal. **f** Kaplan-Meier survival curve following IT administration of an intermediate (10^{11} vg per mouse) dose of AAV-mSPB + BLES with dox removed 7 days after vector delivery. This survival curve is for the entire duration of the experiment up until manuscript submission from Fig. 6f. **g** Mean weight measurements with SD from **(f)** shows that mice administered AAV-mSPB demonstrates a similar increase in weight as mice on dox for nearly 200 days. (All survival curve P values = 2-tailed Log-rank, Mantel-Cox test with significance set at $P<0.05$, except **(f)** = 2-tailed Log-rank, Mantel-Cox test with Bonferroni correction, significance is set at $P=0.0167$; ns = not significant).

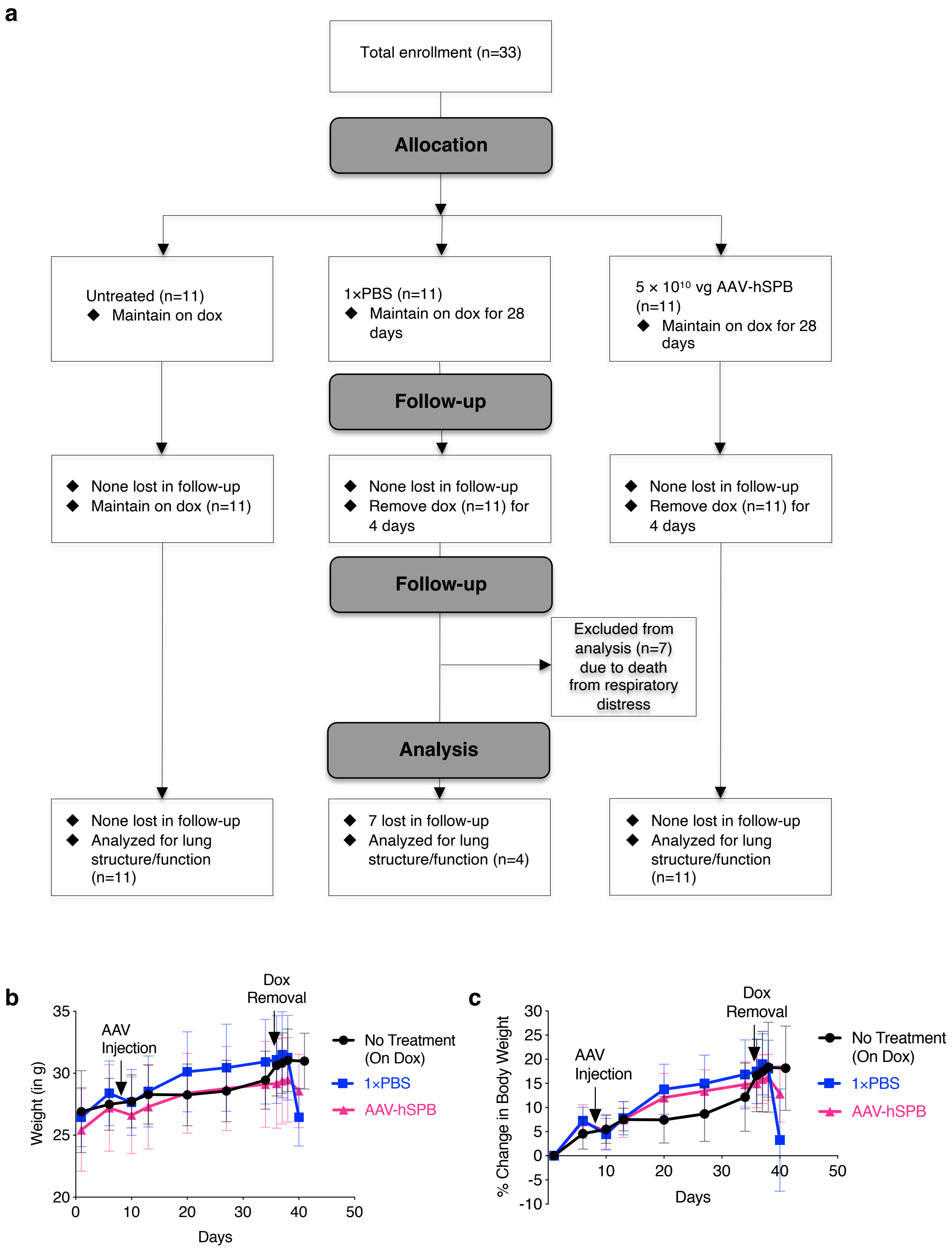
a
Total enrollment (n=33)
Allocation
Untreated (n=11)
◆ Maintain on dox
1×PBS (n=11)
◆ Maintain on dox for 28 days
5 × 10[10] vg AAV-hSPB (n=11)
◆ Maintain on dox for 28 days
Follow-up
◆ None lost in follow-up
◆ Maintain on dox (n=11)
◆ None lost in follow-up
◆ Remove dox (n=11) for 4 days
◆ None lost in follow-up
◆ Remove dox (n=11) for 4 days
Follow-up
Excluded from analysis (n=7) due to death from respiratory distress
Analysis
◆ None lost in follow-up
◆ Analyzed for lung structure/function (n=11)
◆ 7 lost in follow-up
◆ Analyzed for lung structure/function (n=4)
◆ None lost in follow-up
◆ Analyzed for lung structure/function (n=11)
b
Weight (in g)
Days
AAV Injection
Dox Removal
No Treatment (On Dox)
1×PBS
AAV-hSPB
c
% Change in Body Weight
Days
AAV Injection
Dox Removal
No Treatment (On Dox)
1×PBS
AAV-hSPB

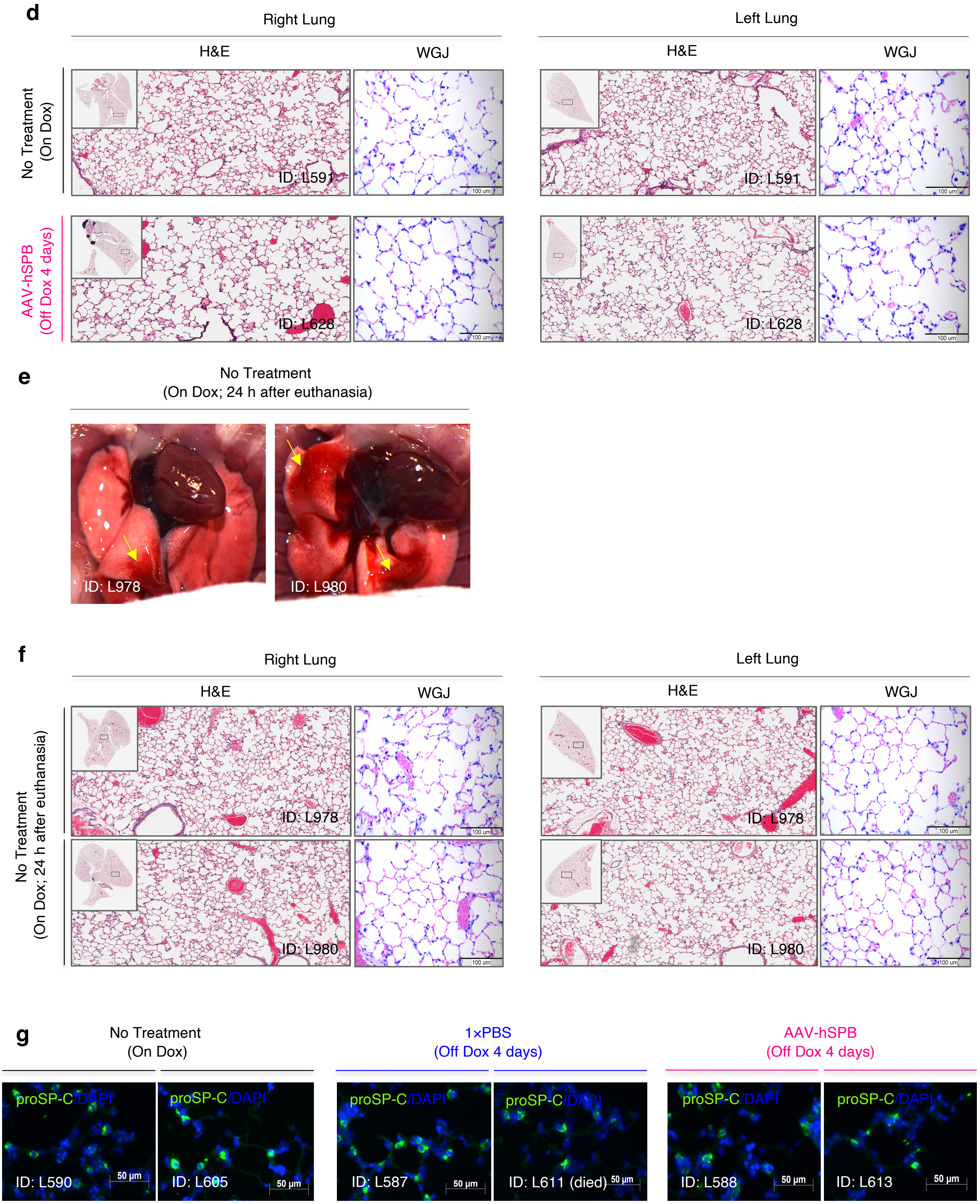
d
Right Lung
Left Lung
H&E
WGJ
H&E
WGJ
No Treatment
(On Dox)
AAV-hSPB
(Off Dox 4 days)
ID: L591
ID: L591
ID: L628
ID: L628
100 um
e
No Treatment
(On Dox; 24 h after euthanasia)
ID: L978
ID: L980
f
Right Lung
Left Lung
H&E
WGJ
H&E
WGJ
No Treatment
(On Dox; 24 h after euthanasia)
ID: L978
ID: L978
ID: L980
ID: L980
100 um
g
No Treatment
(On Dox)
1×PBS
(Off Dox 4 days)
AAV-hSPB
(Off Dox 4 days)
proSP-C/DAPI
ID: L590
ID: L605
ID: L587
ID: L611 (died)
ID: L588
ID: L613
50 µm

h

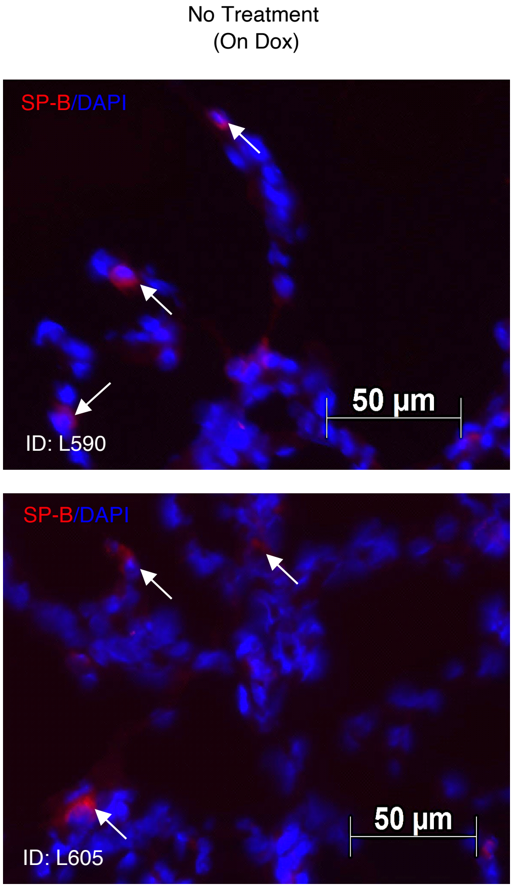
No Treatment
(On Dox)
SP-B/DAPI
50 µm
ID: L590
SP-B/DAPI
50 µm
ID: L605

i

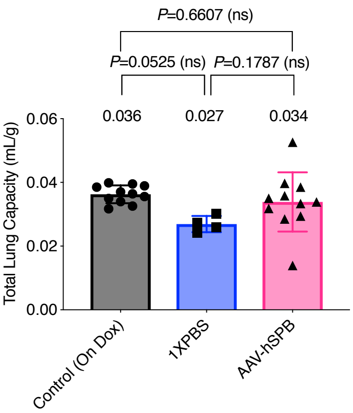
P=0.6607 (ns)
P=0.0525 (ns)
P=0.1787 (ns)
0.036
0.027
0.034
Total Lung Capacity (mL/g)
0.06
0.04
0.02
0.00
Control (On Dox)
1XPBS
AAV-hSPB

j

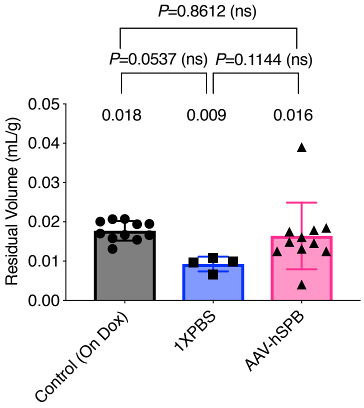
P=0.8612 (ns)
P=0.0537 (ns)
P=0.1144 (ns)
0.018
0.009
0.016
Residual Volume (mL/g)
0.05
0.04
0.03
0.02
0.01
0.00
Control (On Dox)
1XPBS
AAV-hSPB

k

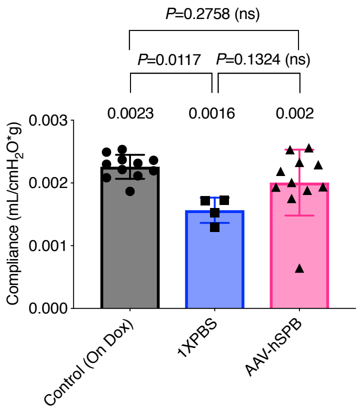
P=0.2758 (ns)
P=0.0117
P=0.1324 (ns)
0.0023
0.0016
0.002
Compliance (mL/cmH2O*g)
0.003
0.002
0.001
0.000
Control (On Dox)
1XPBS
AAV-hSPB

l

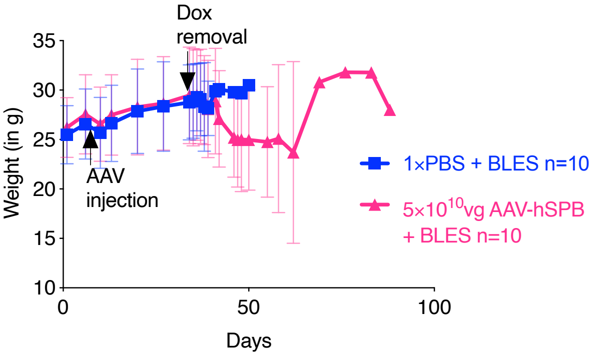
Dox
removal
AAV
injection
Weight (in g)
35
30
25
20
15
10
0
50
100
Days
1×PBS + BLES n=10
5×10^10vg AAV-hSPB
+ BLES n=10

m

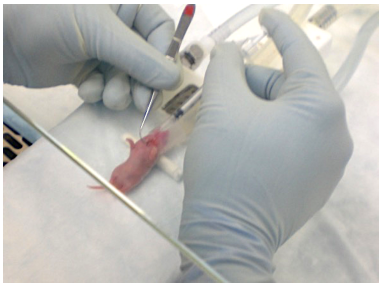

n

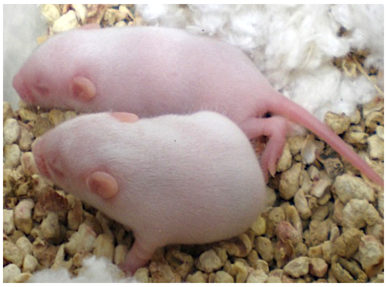

o

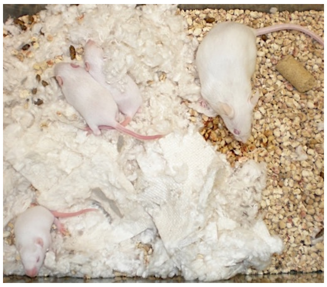

Supplementary Fig. 9 IT administration of 5 × 10^{10} vg per mouse or 10^{11} vg per mouse of AAV6.2FF human *proSFTPB* cDNA (AAV-hSPB) prevents lung damage and improves median survival in both adult and neonatal SP-B deficient mice. a Consort diagram for the structure and function study from Fig. 7. **b** Mean weight measurements with SD from all surviving mice in Fig. 7. **c** Percent change in mean body weight with SD from all surviving mice in Fig. 7 (For both **(b)** and **(c)**, n=11 per group at all timepoints until Dox removal where 1×PBS n=4). **d** Representative H&E and WGJ staining of paraffin embedded whole right and left lungs 4 days after dox removal from a mouse on dox, and one treated with AAV-hSPB (H&E Scale bar, 200 µm; WGJ Scale bar, 100 µm). **e** Gross lung images from transgenic SP-B mice on dox euthanized 24 h before lung collection. This experiment was conducted to demonstrate that the severe lung phenotype in an SP-B deficient mouse that was found dead in Fig. 7f (ID: L609) was not caused by post-mortem decomposition, but rather the lack of SP-B. The gross lung images demonstrate less discoloration and injury than in the 1×PBS mice off dox found dead in Fig. 7f. Yellow arrows indicate regions of discoloration. **f** H&E and WGJ staining of paraffin embedded whole right and left lungs from **(e)** 24 h after euthanasia (H&E Scale bar, 200 µm; WGJ Scale bar, 100 µm). Histological lung injury is minimal or absent compared to the 1×PBS mouse off dox found dead in Fig. 7f (ID: L609). **g** Epifluorescence images of proSP-C (green) and DAPI (blue) from frozen lung sections of: SP-B deficient mice on dox (n=2); 1×PBS plus BLES treated SP-B deficient mice off dox for 4 days (n=2); and 5 × 10^{10} vg AAV-SPB plus BLES treated SP-B deficient mice off dox for 4 days (n=2) (Scale bar, 50 µm). **h** Epifluorescence images of SP-B (red) and DAPI (blue) from frozen lung sections of SP-B deficient mice on dox (n=2) (Scale bar, 50 µm). **i** Total Lung Capacity corrected for body weight (in mL per g). **j** Residual Volume corrected for body weight (in mL per g). **k** Compliance corrected for body weight (in mL per cmH_2O per g). All lung function data (**i-k**) are from SP-B deficient mice on dox; 1×PBS plus BLES off dox for 4 days; and 5 × 10^{10} vg AAV-SPB plus BLES off dox for 4 days, and are presented as the mean with SD (n=11 except 1×PBS plus BLES n=4). (All lung function *P* values = ordinary one-way ANOVA with Tukey's multiple comparisons post hoc test; ns = not significant). **l** Mean weight measurements with SD of all surviving mice treated with either 1×PBS or 5 × 10^{10} vg per mouse (low dose) from Fig. 8a. **m** Image of an intratracheal injection (tracheotomy) of AAV-hSPB plus BLES into a P8 pup from Figs. 8c-e. **n** P8 pups 5 hr post-surgery from Figs. 8c-e. **o** P10 pups 24 hr post surgery with their mother from Figs. 8c-e.

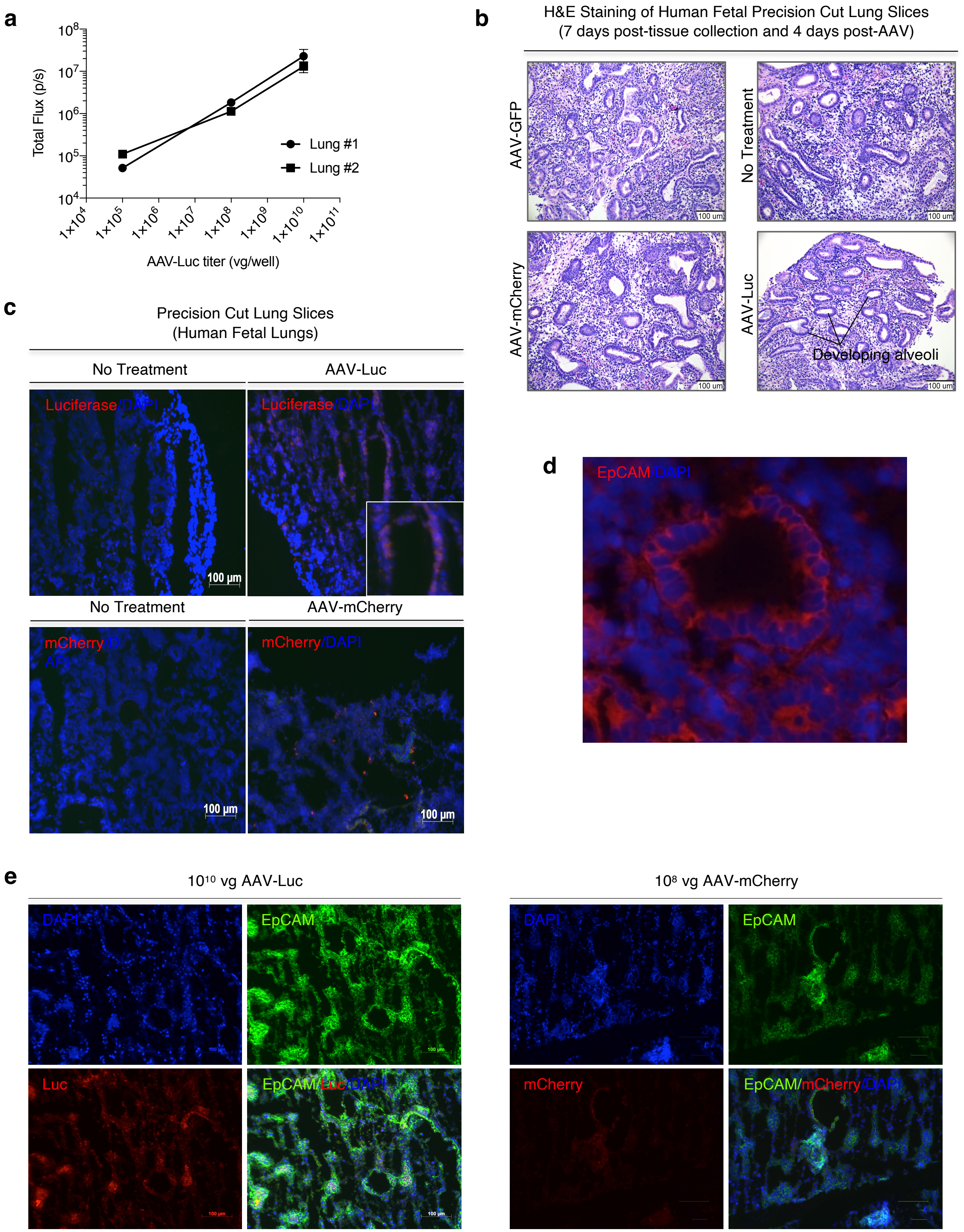
a
Total Flux (p/s)
10^8
10^7
10^6
10^5
10^4
Lung #1
Lung #2
1x10^4
1x10^5
1x10^6
1x10^7
1x10^8
1x10^9
1x10^10
1x10^11
AAV-Luc titer (vg/well)
b
H&E Staining of Human Fetal Precision Cut Lung Slices
(7 days post-tissue collection and 4 days post-AAV)
AAV-GFP
No Treatment
AAV-mCherry
AAV-Luc
Developing alveoli
100 um
c
Precision Cut Lung Slices
(Human Fetal Lungs)
No Treatment
AAV-Luc
Luciferase/DAPI
100 μm
No Treatment
AAV-mCherry
mCherry/DAPI
d
EpCAM/DAPI
e
10^10 vg AAV-Luc
10^8 vg AAV-mCherry
DAPI
EpCAM
Luc
EpCAM/Luc/DAPI
mCherry
EpCAM/mCherry/DAPI

Supplementary Fig. 10 Precision Cut Lung Slices (PCLS) from human fetal lungs (Gestational ages: 16^{+0} and 16^{+3} weeks) transduced with AAV6.2FF vectors. a Quantification of bioluminescence from the IVIS image in Fig. 10c as total flux (photons per second). Total flux of AAV-Luc wells are represented as the mean with SD (n=2 fetuses; 2 lung slices per fetus per dose for AAV-Luc). **b** Representative H&E images from human fetal PCLS that were fixed in 4% PFA immediately after IVIS imaging in Fig. 10c (Scale bar, 100 µm). The lungs demonstrate established acinar structures, and based on the age of the fetuses, should be in the late pseudoglandular stage of lung development. **c** Representative epifluorescence images of luciferase (red), mCherry (red), and DAPI (blue) in frozen PCLS 4 days after transduction with 10^8 vg per well of AAV-Luc or AAV-mCherry (Scale bar, 100 µm). **d** Representative epifluorescence image of EpCAM (red) and DAPI (blue) from frozen PCLS sections demonstrating the ubiquitous presence of epithelial cells in the developing alveoli. **e** Representative epifluorescence images of DAPI (blue), EpCAM (green), and luciferase (red) or mCherry (red) in frozen PCLS 4 days after transduction with 10^{10} vg per well of AAV-Luc or 10^8 vg per well of AAV-mCherry (Scale bar, 100 µm).
